# Dynamic network interactions among distinct brain rhythms as a hallmark of physiologic state and function

**DOI:** 10.1038/s42003-020-0878-4

**Published:** 2020-04-27

**Authors:** Aijing Lin, Kang K. L. Liu, Ronny P. Bartsch, Plamen Ch. Ivanov

**Affiliations:** 1 Department of Mathematics, School of Science, Beijing Jiaotong University, Beijing, 100044 China; 2 Keck Laboratory for Network Physiology, Department of Physics, Boston University, Boston, MA 02215 USA; 3 Department of Neurology, Beth Israel Deaconess Medical Center, Harvard Medical School, Boston, MA 02115 USA; 4 Department of Physics, Bar-Ilan University, Ramat Gan, 5290002 Israel; 5 Division of Sleep Medicine, Brigham and Women’s Hospital, Harvard Medical School, Boston, MA 02115 USA; 6 Institute of Solid State Physics, Bulgarian Academy of Sciences, Sofia, 1784 Bulgaria

**Keywords:** Sleep, Circadian rhythms and sleep

## Abstract

Brain rhythms are associated with a range of physiologic states, and thus, studies have traditionally focused on neuronal origin, temporal dynamics and fundamental role of individual brain rhythms, and more recently on specific pair-wise interactions. Here, we aim to understand integrated physiologic function as an emergent phenomenon of dynamic network interactions among brain rhythms. We hypothesize that brain rhythms continuously coordinate their activations to facilitate physiologic states and functions. We analyze healthy subjects during sleep, and we demonstrate the presence of stable interaction patterns among brain rhythms. Probing transient modulations in brain wave activation, we discover three classes of interaction patterns that form an ensemble representative for each sleep stage, indicating an association of each state with a specific network of brain-rhythm communications. The observations are universal across subjects and identify networks of brain-rhythm interactions as a hallmark of physiologic state and function, providing new insights on neurophysiological regulation with broad clinical implications.

## Abstract (web summary)

Lin & Liu et al. analyze EEG recordings and report that each sleep stage is characterized by a specific ensemble of coupling profiles and a network of brain-rhythm communications. Their work challenges the current paradigm of understanding physiologic states through distinct or dominant brain rhythms and identifies networks of brain-rhythm interactions as a signature of physiologic state and function.

## Introduction

At the integrated systems level, brain dynamics are characterized by distinct rhythms^1^ with complex spatio-temporal behaviors over multiple levels of organization and broad range of timescales^2–4^. At the microscopic level, complex firing patterns of individual neurons and communications between neuronal populations across brain areas lead to the emergence of distinct brain rhythms^5,6^ with different frequency characteristics and spatio-temporal dynamics^7,8^. At the integrated macroscopic level, brain rhythms have been associated with a range of physiologic states^9–11^, various neurophysiological and cognitive functions^12–16^, and pathological conditions^17–20^. The traditional paradigm in brain research focuses on exploring the role of brain rhythms and their association with specific physiologic states and functions^1,21^, which are often considered mutually exclusive^22^. For example, low-frequency delta waves dominate during deep sleep, while high frequency alpha and beta waves are prevalent during wakefulness^1,10,22^. Moreover, the coexistence of brain rhythms corresponding to opposite physiologic functions (such as alpha and delta waves) has been related to pathological conditions^20^. Thus, research in the field has primarily focused on individual brain rhythms and specific pairwise interactions to understand the complexity in their temporal dynamics, neuronal origins, and spatial distribution across brain areas^7,8,17,23^. This traditional approach is further motivated by observations of (i) quasi-steady-state behavior of brain rhythms at large timescales within each physiologic state, and (ii) gradual change in the amplitude of individual brain rhythms with transitions from one physiologic state to another^1,10,24^. In this classical paradigm of defining physiologic states and functions by the presence of dominant brain rhythms, little attention is paid to the dynamics of nondominant rhythms. While recent studies have identified coupling between certain brain rhythms^25,26^, it remains unknown whether dominant and nondominant brain rhythms dynamically interact, and how the network of brain-wave interactions relates to physiologic states.

Here, we study physiologic function through the collective behavior of dynamical network interactions among multiple brain rhythms. We hypothesize that not only the specific temporal and spatial characteristics of individual brain rhythms but also their coordinated interactions play important role in generating and maintaining physiologic states and functions. Further, we hypothesize that the short-term modulations in the amplitude of brain rhythms, that occur on top of their quasi-steady-state behavior at large timescales, carry important information about their communications.

In this study, we address the fundamental question of how different brain rhythms continuously interact and collectively behave as a network to facilitate distinct physiologic states and integrated physiologic functions. We analyze temporal patterns in the amplitude of brain waves activation, and probe for coordination and synchronous modulation in dominant and nondominant brain rhythms. We demonstrate the presence of robust coupling profiles representing dynamical network interactions among brain rhythms. We discover an entire ensemble (“alphabet”) of key profiles of brain-wave interactions, which are universally observed for different brain areas and across subjects. Moreover, we find that these interaction profiles and the related networks change with transition from one physiologic state to another, and thus, are a unique signature of physiologic state and function.

## Results

### Synchronous modulation in the amplitudes of brain rhythms

**Fig. 1:**
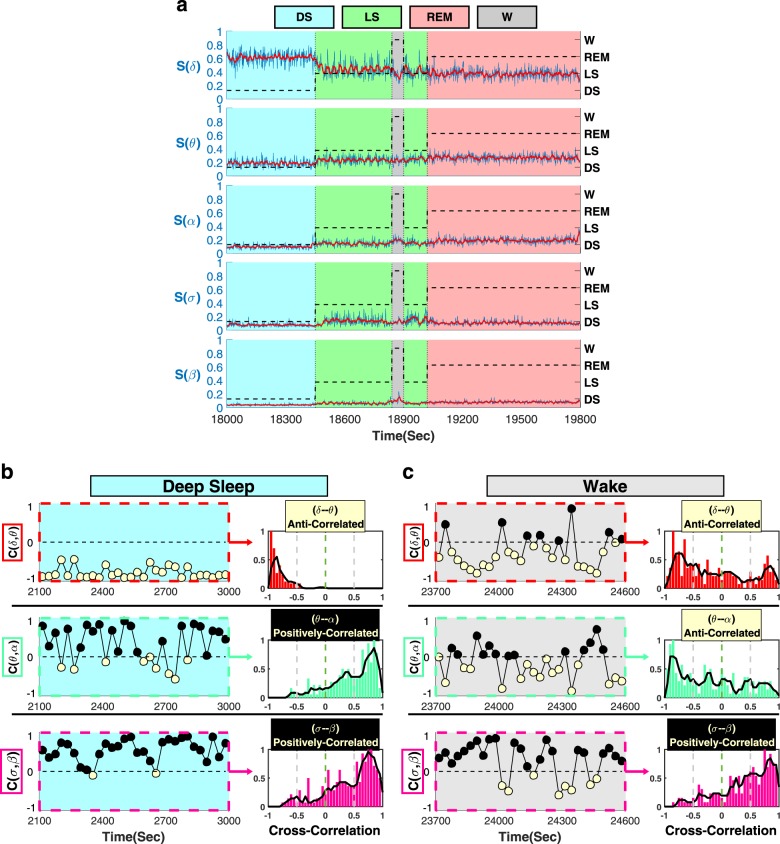
Complex temporal dynamics and distinct profiles of brain wave interactions. **a** Normalized spectral power of brain rhythms (Methods) at the central C3 EEG channel from a healthy subject during sleep in 1-s time resolution (blue lines). Red lines show moving average (window 14 s; step 1 s) and represent short-term modulation in the activation of brain rhythms. Black dashed lines represent sleep stages. Time series of spectral powers in (**a**) are shown for a typical 30 min segment of EEG recording when the subject undergoes a transition from deep sleep (DS) to light sleep (LS), interrupted by a brief wake stage/arousal (W), and then to REM. Data are consistent with traditional description of different sleep stages—dominant *δ* power and minimal *α* during DS; significant decrease in *δ* and increase in *α* power with transition from DS to LS and W; *σ* waves activation during LS (Supplementary Fig. 1). Observations at smaller timescales reveal a continuous modulation in the spectral power on top of the quasi-steady-state brain-wave behavior observed at large scales within each sleep stage, raising the hypothesis that brain waves dynamically interact with each other. We find a specific profile for each pair of brain-wave interactions typical for each sleep stage. Cross-correlation *C* between the spectral power of different brain rhythms is calculated in non-overlapping windows of 30 s during a 15-min deep-sleep episode (**b**) and a 15-min wake episode (**c**). Three classes of brain-wave interactions are observed: stable anti-correlations with *C* < 0 (*δ*–*θ*, yellow symbols); stable positive correlations, *C* > 0 (*σ*–*β*, black symbols); transition from positive- to anti-correlation (*θ*–*α*). Distribution of cross-correlation values for three pairs of brain-wave interactions obtained from the same subject during all DS episodes (**b**) and all wake episodes (**c**) over the entire night. Each pair is characterized by a specific profile—values *C* > 0 correspond to positive correlation and *C* < 0 to anti-correlation. These observations reveal brain-wave interactions with specific profiles that are characteristic of physiologic states.

To identify and quantify temporal interactions between brain rhythms, we analyze EEG spectral power in five different physiologically relevant frequency bands (*δ*, *θ*, *α*, *σ*, and *β*) corresponding to five basic brain waves. Our analyses confirm the classical picture that on large timescales of minutes to hours, physiologic states are characterized by the presence of dominant brain waves and their steady-state behavior—e.g., dominant *δ* wave and minimal *α* wave activity in deep sleep; gradual decrease in *δ* power during light sleep accompanied by increased *σ* and *θ* power, and elevation in *α* rhythm during REM and arousals/wake (Fig. 1a; Supplementary Fig. 1). However, a closer inspection at much shorter timescales of seconds reveals that within each physiologic state all brain waves exhibit continuous fluctuations and complex temporal patterns around their respective steady-state behavior. This raises the hypothesis that interactions among brain rhythms may be encoded in continuous coordinated modulations of their relative spectral power. Thus, to probe dynamical interactions between different brain rhythms, we first derive the normalized spectral power of each brain wave in 1 s resolution, and we calculate the cross-correlation between each pair of brain rhythms within short-time windows of 30 s (“Methods”, Supplementary Fig. 2).

Analyzing EEG recordings from a healthy subject during 8 h of night-time sleep^27,28^, we discover strong dynamical interactions between different brain waves, and that these interactions are mediated through complex fluctuations and coordinated modulation in the spectral power of the individual brain waves. Specifically, we find that these interactions are characterized by high degree of temporal cross-correlations, and that different pairs of brain waves are characterized by different cross-correlation patterns. For example, a strong positive cross-correlation is consistently observed for the *σ*–*β* brain wave interaction, indicating a remarkably synchronous behavior at short timescales, where increase (decrease) in the amplitude of one brain wave is accompanied by a corresponding parallel increase (decrease) in the amplitude of the other brain wave (Fig. 1b, c). In contrast, we find that the *δ*–*θ* interaction is characterized by a strong anti-correlation that remains stable in time (Fig. 1b, c), indicating that the amplitude modulation of one brain wave is in the opposite direction with respect to the amplitude modulation of the other brain wave. These observations demonstrate a remarkable complexity in brain rhythms interactions, as different brain waves exhibit markedly different patterns of cross-correlation.

**Fig. 2:**
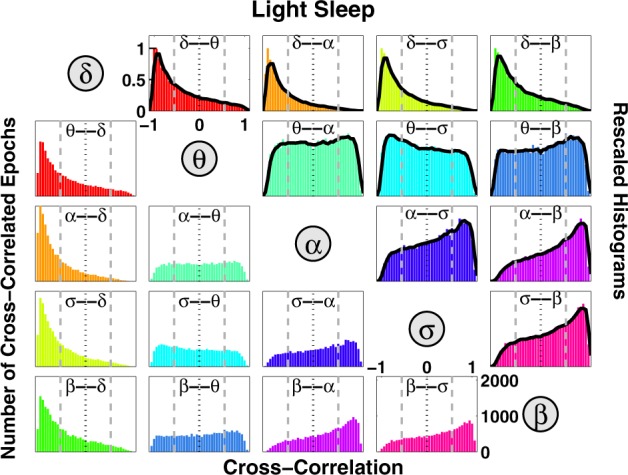
Alphabet of brain-wave interactions as a signature of physiologic state. Distributions of cross-correlation for different pairs of brain waves. Distributions are obtained from the cross-correlation time series values for each pair of brain-wave interactions (Fig. 1) pooled from all subjects during all light sleep (LS) episodes over the entire night-time sleep period. Since cross-correlation values are obtained for consecutive 30-s segments of the recordings (“Methods”), each distribution represents the number of cross-correlated epochs (*y*-axis) for a given cross-correlation value (*x*-axis), as shown in the panels below the diagonal line. Different colors correspond to different pairs of cross-correlated brain waves. Distributions rescaled to their respective maximum value are shown in the panels above the diagonal line, where solid black line in each panel represents a moving average of the rescaled histogram (smoothed profile). Profiles skewed to the left with peak at *C* < 0 indicate anti-correlated pairs of brain waves, while profiles skewed to the right with peaks at *C* > 0 indicate positively correlated brain-wave interactions. Each pair of brain-wave interactions is characterized by a distinct profile of the rescaled histogram, indicating that besides the quasi-steady-state behavior of brain rhythms observed at large timescales during a given physiologic state (Supplementary Fig. 1), there are complex dynamics of continuous communications among brain rhythms that occur at shorter timescales. The ensemble of these profiles forms a specific “alphabet” representing brain-wave interactions.

Further, we ask the question whether brain-wave interactions change from one physiologic state to another. We discover that certain pairs of brain waves show a pronounced transition in their cross-correlation—e.g., *θ*–*α* interaction exhibits strong positive cross-correlation during deep sleep (Fig. 1b), and undergoes a transition to anti-correlated interaction during wake (Fig. 1c). Such dramatic change in the interaction pattern between brain waves with transition from one physiologic state to another reveals an intriguing interplay between brain-rhythm communications and physiologic states—i.e., on one hand, changes in the mechanism of physiologic regulation impact the coordinated activation of different brain rhythms (Fig. 1), while on the other hand, a particular mosaic of dynamic patterns of brain-rhythm interactions may uniquely define each physiologic state (Fig. 2).

### Coupling profiles of brain-rhythm interactions

To explore the association between brain-rhythm coordination and physiologic states, and to test whether brain-wave interaction patterns are consistent across subjects, we next systematically analyze a group of 34 healthy young subjects (17 female and 17 male, see “Methods”), and we probe for interactions between all pairs of brain waves. We find that during a specific physiologic state (e.g., light sleep), each pair of brain waves exhibits a distinct pattern for the distribution of cross-correlation values *C* (Fig. 2). Distribution profiles skewed to the left with peak at *C* < 0, as observed for the *δ–θ, δ–α, δ–σ, δ–β* pairs, indicate strong anti-correlated brain-wave interactions; in contrast, profiles skewed to the right with peaks at *C* > 0, as observed for *α–σ, α–β, σ–β* pairs, indicate strong positive correlations. Other pairs such as *θ–α, θ–σ, θ–β* exhibit more homogeneous cross-correlation distribution patterns, indicating a different mechanism of brain-wave coordination (Fig. 2). Remarkably, the entire ensemble of brain-wave interaction patterns is consistent when comparing all subjects in our database during the same physiologic state, indicating a universal mechanism underlying brain-rhythm interactions (Supplementary Fig. 3). These observations reveal that at short timescales, there is a previously unrecognized complex organization among brain rhythms which continuously coordinate and synchronize their dynamics. For a given physiologic state, this organization among brain rhythms is manifested by a unique ensemble of distinct types of interaction patterns (cross-correlation profiles): (i) pairs of brain waves that exhibit strong positive correlations with parallel modulation in their amplitude of activation; (ii) pairs of brain waves that are strongly anti-correlated, where one brain wave adjusts its relative amplitude in the opposite direction in response to changes in the dynamics of the other wave; (iii) pairs of brain waves that, within a given physiologic state, exhibit transient behavior in their interactions, leading to a more homogeneous cross-correlation distribution (Fig. 2).

These distinct types of reciprocal brain-wave relations are universal across subjects and indicate that interactions between different pairs of brain rhythms play different roles in physiologic regulation. Our findings demonstrate the need to extend the traditional framework of understanding physiologic states through the prism of distinct or dominant brain rhythms and their homeostatic steady-state dynamics at large timescales^10,24^. In addition to this classical picture, we find that for a given physiologic state, there is a unique “alphabet” of brain-rhythm communications (Fig. 2) embedded in transient brain wave modulations at short timescales.

### Alphabet of coupling profiles as hallmark of sleep stages

**Fig. 3:**
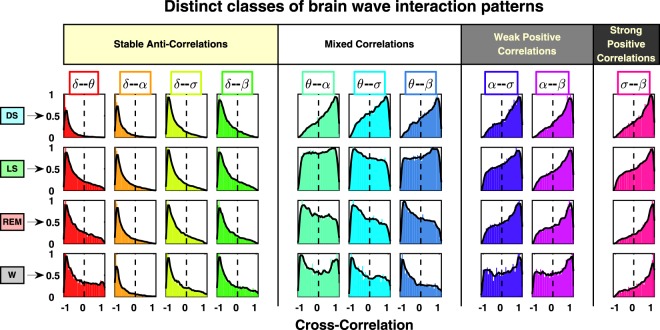
Transitions in brain-wave interactions across physiologic states. Profiles of cross-correlation distributions for each pair of brain waves during different physiologic states (sleep stages) obtained by pooling data from all subjects. Colors correspond to different pairs of brain waves, as shown in Fig. 2. To better visualize the different profiles of the cross-correlation distributions, all histograms are rescaled so that the peak value is one. Solid black line in each panel represents a moving average of the rescaled histogram (smoothed profile). Each physiologic state (horizontal row) is characterized by a specific set of profiles representing the alphabet of interactions for different pairs of brain waves. Further, with transition from one physiologic state to another (vertical column), the cross-correlation profile for each pair of brain waves changes, leading to a reorganization of the ensemble of brain-wave interactions. Three classes of brain-wave interactions are observed with transitions across physiologic states: (i) a class of pronounced anti-correlations as represented by distribution profiles with a peak at cross-correlation *C* < 0 (e.g., *δ*–*θ*, *δ*–*σ*, *δ–α, δ–β)*; (ii) pronounced positive correlations as represented by distribution profiles with a peak at cross-correlation *C* > 0 (e.g., *α*–*σ*, *α*–*β*, *σ*–*β*); (iii) pairs of brain waves that are characterized by a transition from positive- to anti-correlated interactions (e.g., *θ*–*α*, *θ*–*σ*, *θ*–*β*). These observations indicate a complex hierarchical organization in the way brain rhythms communicate with each other to facilitate distinct physiologic states and functions, and that a specific set of brain wave interactions (alphabet of profiles) underlies each physiologic state.

Next, we investigate the association between the alphabet of brain-wave interactions and different physiologic states. For each pair of brain rhythms, we track how the cross-correlation distribution profile changes with transition from one sleep stage to another. Comparing profiles of brain-wave interactions for different physiologic states, we discover that each state is characterized by a specific ensemble of profiles, as shown in Fig. 3 by panels along the horizontal direction. These state-dependent ensembles of profiles are robust as they are consistently observed in all individual subjects (Supplementary Fig. 3 and statistical tests in “Methods”) during the same sleep stage, thus reflecting a fundamental feature of physiologic states. We also investigate how the ensemble of interaction profiles reorganizes with transitions across sleep stages. We find that with transition from one sleep stage to another (vertical direction in Fig. 3), the cross-correlation profile for each pair of brain waves changes, leading to a complex reorganization of the entire alphabet of brain-wave interactions. Based on how the shape of each interaction profile evolves across sleep stages, we identify three major classes of brain-wave interactions: (i) a class of brain rhythms that exhibit stable negative cross-correlations (e.g., *δ*–*θ*, *δ*–*σ*, *δ*–*α*, *δ*–*β*); (ii) brain rhythms that interact through stable positive cross-correlations (e.g., *α*–*σ*, *α*–*β*, *σ*–*β*); and (iii) a class of brain-wave interactions that are sleep-stage dependent and undergo a pronounced transition from positive to negative cross-correlation with transition from DS to LS, REM, and wake (e.g., *θ*–*α*, *θ*–*σ*, *θ*–*β*).

Further, we track the evolution of the interaction profiles within each class of brain rhythm communications, and we identify distinct subsets: (i) in the class of positively correlated pairs of brain waves, *α–σ* and *α–β* interactions exhibit gradual reduction in the degree of cross-correlations with transition from DS to LS, REM, and wake, as indicated by the shift of the distribution profiles from *C* > 0 to *C* < 0 values. In contrast, *σ*–*β* interactions exhibit gradual increase in positive cross-correlations (Fig. 3); (ii) in the class of anti-correlated pairs of brain waves, *δ*–*θ* interactions exhibit gradual decrease in anti-correlations (fatter tails with *C* > 0 for REM and wake, Fig. 3), whereas *δ*–*α* interactions exhibit pronounced anti-correlation that remains stable for all sleep stages. Notably, these interaction profiles result from short-scale synchronous modulation in brain-wave amplitudes, and are masked, due to shifts in global EEG power, when absolute spectral power (instead of relative power) is considered for each frequency band (“Methods”, Supplementary Fig. 4). The observed classes and subsets of brain wave interactions indicate a hierarchical reorganization of the entire ensemble of interaction profiles with transition across physiologic states. These findings indicate a direct association between patterns of coordination among distinct brain rhythms at short timescales and integrative physiologic function at longer timescales.

### Degree of coupling strength in brain-rhythms interaction

**Fig. 4:**
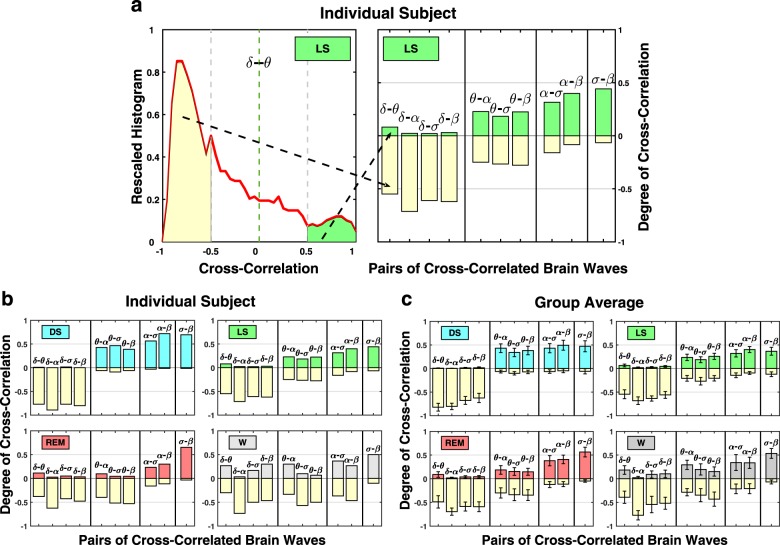
Robust sleep-stage stratification in the degree of cross-correlation for different pairs of brain rhythms. **a** Degree of cross-correlation as a measure of brain-rhythm interactions calculated as fraction of total time during a particular physiologic state when significant cross-correlations are observed (significance threshold |*C*| = 0.5; Supplementary Fig. 6). (left panel) The degree of positive cross-correlation between *δ* and *θ* waves during LS corresponds to the ratio of the distribution area for *C* > 0.5 and the total area under the distribution curve; the degree of *δ*–*θ* anti-correlation during LS equals the ratio of the distribution area for *C* < −0.5 out of the total distribution area under the red line. The degree of positive correlations for pairs of brain-wave interactions is plotted as positive bars shaded in same color as the corresponding physiologic state, whereas the degree of anti-correlation is represented by negative yellow bars. Bar charts in (**b**, **c**) quantify the difference between profiles of the cross-correlation distributions shown in Fig. 3. Three major classes of brain-wave interactions are observed for all sleep stages: (i) strongly anti-correlated (negative bars); (ii) strongly positively correlated (positive bars); (iii) pairs of brain waves that switch from positively- to anti-correlated interactions with transitions across sleep stages. Within these classes, certain pairs of brain waves exhibit different trends in the nature of their interactions: in the class of positively correlated pairs, *α*–*σ* and *α*–*β* interactions exhibit a reduction in positive correlations (decreasing height of positive bars) with transition from DS to LS, REM, and wake, while *σ*–*β* interactions exhibit gradual increase in positive correlations (Fig. 3). In the class of anti-correlated pairs, *δ*–*θ* interactions exhibit decrease in anti-correlations, whereas *δ*–*α* interactions exhibit stable anti-correlation for all sleep stages. This stratification pattern in brain-wave interactions is consistently observed for individual subjects (**b**), as well as in the group average (**c**). Error bars represent group standard deviation. This robust sleep-stage stratification pattern reveals a universal organization in brain-wave interactions.

To characterize the different classes of brain-wave interaction profiles as well as the hierarchical reorganization of the entire ensemble of profiles across sleep stages, we introduce a measure to quantify the degree of cross-correlation between all pairs of brain rhythms. The measure is defined as the fraction of time when significant positive or negative cross-correlation values are observed during all episodes of a specific sleep stage throughout the night (Fig. 4). This measure is closely associated with the occurrence of short-scale transient physiologic events within a physiologic state that can not be identified at longer timescales (Supplementary Figs. 1 and 2). Since each brain-wave interaction profile in Fig. 3 is represented by the distribution of cross-correlation values *C*, where cross-correlation values are obtained in non-overlapping 30-s windows, calculating the number of *C* values that are above a significance threshold quantifies the fraction of time when significant cross-correlation between two brain waves is observed. We choose a threshold of *C*_0_ = 0.5 (based on surrogate test; “Methods” and Supplementary Fig. 6) to define significant positive cross-correlation values *C* > 0.5, and significant anti-correlation values *C* < −0.5 (for statistical tests, see “Methods” and Supplementary Fig. 7). In each distribution profile, the degree of positive correlation is represented by the area under the profile curve with *C* > 0.5, whereas the degree of anti-correlation corresponds to the area under the profile with *C* < −0.5. Thus, we characterize each interaction profile by the areas corresponding to the two extreme parts of the cross-correlation distribution (Fig. 4a), where the height of each positive or negative bar represents the fraction of the recording during a specific physiologic state with significant positive correlation (*C* > 0.5) or with significant anti-correlation (*C* < −0.5).

This approach allows to quantify the three classes of brain-wave interactions based on differences in their respective profiles (Fig. 4): high degree of positive cross-correlations (shaded positive bars); high degree of anti-correlations (yellow negative bars); and state-dependent interactions that change from positive to anti-correlation with transitions across sleep stages. This measure and graphic representation of the strength of interactions among brain rhythms is used in Fig. 4 to demonstrate different classes of brain wave interactions and to quantify how these interactions change with sleep stages. Moreover, we find a clear sleep-stage stratification pattern imprinted on the different classes of the brain wave interactions (Fig. 4), which is robust in respect to the cross-correlation threshold *C*_0_ (“Methods”, Supplementary Fig. 8). This finding indicates that brain rhythms collectively adjust and coordinate their activation in response to changes in physiologic regulation during different sleep stages. Further, the same sleep-stage stratification pattern of brain-wave interaction is consistently observed for each individual subject as well as for the group average (Fig. 4), indicating a universal mechanism underlying communications among brain rhythms. The consistency in brain-wave coupling and universality of sleep-stage stratification among subjects is supported by inferential statistics^29,30^ for the results expected from a larger database (“Methods”, Supplementary Figs. 12 and 13).

We also test whether the reported classes of brain-wave interactions (Fig. 3) and the associated sleep-stage stratification patterns (Fig. 4) are general or depend on cortical location. To that end, we analyze the data derived from different EEG channels (Fp1, Fp2, C3, C4, O1, O2) representing different cortical areas. We find a remarkable consistency in the classes of brain-wave interactions as well as in their sleep-stage stratification when comparing frontal, central and occipital areas within each hemisphere and between the left and right hemisphere (Supplementary Fig. 9).

To demonstrate validity of the results and the relation to underlying physiology, we performed additional tests. To confirm the physiological origin of the observed brain-wave interaction patterns (Figs. 3 and 4), we performed a surrogate test where we analyzed pairs of brain-wave spectral power signals taken from different subjects during the same sleep stage. While spectral power signals from real data exhibit short-term amplitude modulations on top of their quasi-steady-state spectral power level related to the specific sleep stage, these amplitude modulations would not be synchronized for different subjects, and thus, would not result in consistent brain-wave interaction profiles. Indeed, our analyses of such surrogate pairs of brain waves, where the original characteristics of the individual signals are preserved but their amplitude modulations are not synchronized, yield uniform distributions of the Pearson cross-correlation coefficients for all pairs of brain waves and all sleep stages (Supplementary Fig. 10a) with no differentiation in the degree of cross-correlation among the pairs of brain waves and no sleep-stage stratification (Supplementary Fig. 10b). This test confirms that the reported classes of brain wave interaction profiles and their stratification across sleep stages represent real physiologic interactions revealed by our method. To further test the physiological significance of the brain-wave interaction profiles, we performed Fourier phase randomization of the EEG signal which preserves the spectral power in the different frequency bands corresponding to the brain rhythms but eliminates the fine temporal structure in their instantaneous amplitudes. While preserving the ratio between the average spectral power of the different brain waves embedded in the EEG, the procedure eliminates the synchronous modulations of the different frequency bands that underlie brain waves coupling. The results of this surrogate test show very different interaction profiles (Supplementary Figs. 11a and 11b) compared with real data (Figs. 3 and 4). While the class of anti-correlated brain waves is still present for the surrogate data, the shapes of the specific profiles for each pair of brain waves are different compared with real data. Further, the brain-wave interaction profiles in the other three classes (with mixed, weak positive, and strong positive correlations) are dramatically altered. This demonstrates that the reported distinct functional forms of brain-wave coupling represent physiological information related to (i) the relative difference in the total spectral power of each brain wave at large time scale during a given sleep stage, as well as to (ii) the synchronous modulation of brain-wave amplitudes at short timescales.

Because we probe brain-rhythm interactions through short-time scale dynamical patterns in the continuous fluctuations of brain-wave activation, our approach allows to identify and track transient events that occur within each physiologic state. When a particular transient event significantly modulates one brain rhythm (e.g., *δ*-wave) causing a sudden change in its relative power, other brain rhythms (e.g., *θ*, *α*, *σ*, *β*) respond accordingly with reciprocal modulation in their spectral power, yet remaining fairly stable with respect to each other. On one hand, such transient events would be reflected by the emergence of strong anti-correlation between *δ* and all remaining brain rhythms. On the other hand, such events would be also accompanied by strong positive correlations among the remaining brain rhythms. Thus, the measure we introduce to quantify the degree of cross-correlation between brain rhythms (Fig. 4) represents the frequency of occurrence of such transient events, and each pair of brain waves is simultaneously represented by the fraction of time when positive and negative cross-correlation is observed (shaded and yellow bars in Fig. 4).

### Networks of brain-rhythm interactions across sleep stages

**Fig. 5:**
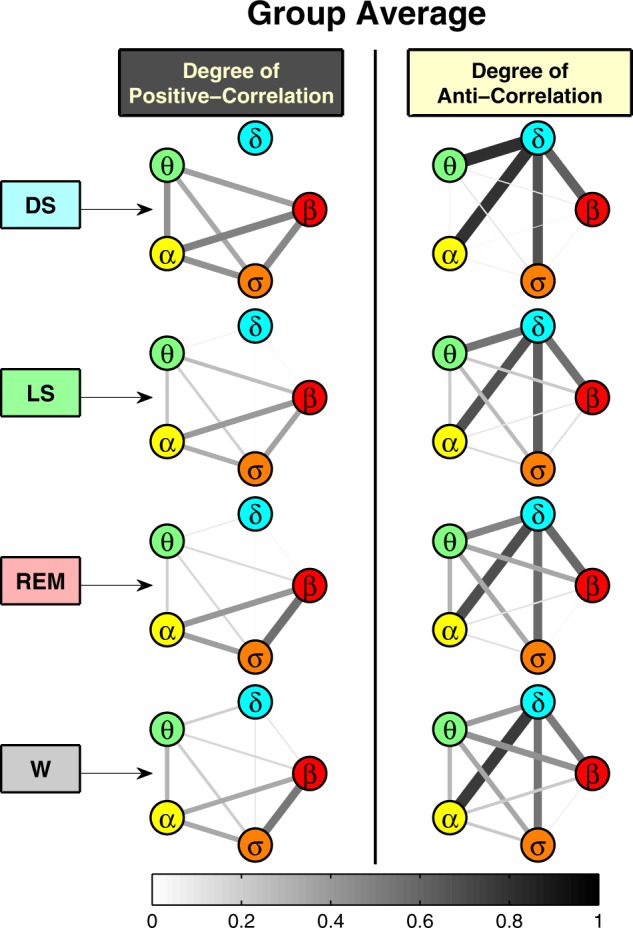
Network communications and topological clustering of brain rhythms. Network representation of coordinated interactions among characteristic brain waves. Network nodes represent brain waves (EEG frequency bands), and network links indicate the degree of cross-correlations for each pair of brain waves (line thickness and darkness correspond linearly to link strength). Two types of networks are shown: left column, where links strength reflects the fraction of time when significant positive correlation (with *C* > 0.5) is found between a given pair of brain waves (as shown by positive bars in Fig. 4); right column, where links strength corresponds to the fraction of time when significant anti-correlation (with *C* < −0.5) is observed (as also shown by negative bars in Fig. 4). During DS, there is a pronounced network cluster of anti-correlated interactions between the *δ* rhythm and all other brain waves, as well as a complementary cluster of only positively correlated interactions between all other brain waves. These networks of coordinated interactions among brain waves evolve with transitions from DS to LS, REM, and wake—the links strength in the anti-correlated cluster between *δ* and all other brain waves decrease, while new positively correlated links emerge. Notably, the *δ*–*α* interaction is always characterized by strong anti-correlations during all sleep stages (no *δ*–*α* link in the positively correlated networks, left column). In contrast, links associated with the *θ* wave (*θ*–*α*, *θ*–*σ*, and *θ*–*β*) that are positively correlated during deep sleep become increasingly anti-correlated during light sleep and REM. Remarkably, the coexistence of both positively- and anti-correlated networks of brain waves interactions within each physiologic state indicates a transient on/off nature of brain-wave communications, where links of different nature can emerge during different periods of time within the same physiologic state. The specific topology and clustering of brain-wave networks during different sleep stages demonstrate a direct association between brain-wave communications and physiologic state and function.

To probe the collective behavior of brain rhythms in relation to physiologic states, we construct networks of positive and anti-correlated interactions, and we track their evolution across sleep stages (Fig. 5). This network approach helps to visualize and dissect brain-wave interactions where positive- and anti-correlated behaviors coexist, and provides a first demonstration of how brain rhythms coordinate collectively as a network to generate distinct physiologic states. During DS, we observe a pronounced network cluster of anti-correlated interactions between the *δ* wave and all other brain waves (Fig. 5). We also identify a coexisting complementary network during DS comprised of only positively correlated interactions between all brain waves except *δ* (Fig. 5). With transition from DS to LS, REM, and wake, the links strength in the anti-correlated cluster between *δ* and all other brain waves decreases, while new positively correlated links emerge indicating a complex reorganization among brain rhythms across physiologic states. We note that links in the positively correlated networks represent parallel coordination of brain waves activation, whereas links in the anti-correlated networks correspond to brain wave interactions of reciprocal and complimentary nature (opposite direction of modulation). Specifically, the *δ*–*α* interaction is always characterized by strong anti-correlation during all sleep stages, and there is no *δ*–*α* link in the positively correlated networks (Fig. 5). This observation is consistent with the traditional understanding of *δ* and *α* waves as the predominant brain rhythms for two opposite physiologic states, i.e., sleep vs. wake. However, the classical description of these physiologic states does not address the nature of *δ*–*α* interaction. Our analyses reveal complex dynamics of reciprocal and competing nature in the coupling between *δ* and *α* waves, which transcends all physiologic states. In contrast to *δ*–*α* interactions, links associated with the *θ* wave (*θ*–*α*, *θ*–*σ*, and *θ*–*β*) that are positively correlated during DS become increasingly anti-correlated during LS and REM, indicating a very different role of *θ*–wave interactions compared with *α*- and *δ*-waves. Further, since network links represent the fraction of time when a certain type of cross-correlation (positive or negative) is observed, the coexistence of both positively- and anti-correlated networks of brain-wave interactions within each physiologic state indicates a very transient on/off nature of brain-rhythms communications, where links of different nature can emerge during different periods of time within the same physiologic state.

The traditional paradigm in brain research focuses on exploring the temporal dynamics and role of individual brain rhythms, and their association with specific physiologic states and functions^1,10^. It is motivated by observations of quasi-steady-state behavior of brain rhythms at large timescales within a given physiologic state (e.g., sleep or wake, sleep stages)^24,31^, and changes in the amplitude (i.e., spectral power) of individual brain rhythms, their synchrony and coherence across cortical areas with transition from one physiologic state to another^32–35^. Our study aims to address the question of how dominant and nondominant brain rhythms dynamically interact. We demonstrate that synchronous short-term modulations in the amplitude of brain rhythms that occur on top of their quasi-steady-state behavior at large timescales, carry important information about the coupling among brain rhythms that are essential characteristics of physiologic state. The presented here approach can detect higher-order interactions among both dominant and nondominant brain rhythms embedded in their fine temporal structure at small timescales (Figs. 3 and 4), and can quantify the change in brain rhythms network communications with transition across distinct sleep stages (Fig. 5). The uncovered coupling forms and network coordination among brain rhythms provide new insights on intrinsic physiologic interactions in the brain.

## Discussion

While neuroscience research has traditionally focused on the firing rates of neurons at the micro scale and the brain areas involved in different tasks at the macroscale, there has been little progress in understanding the computational principles that organize and synthesize lower level signals to produce the effects that we see at larger scales. A number of studies on brain dynamics at the microscopic level have focused on local field potentials^36–40^ and related interactions between neuronal oscillations^41^ often with emphasis on modeling approaches. At the macroscopic integrated system level, studies have traditionally considered coherence of the same cortical rhythm across brain areas^42^ with limited investigations on synchronous occurrence of specific pairs of cortical rhythms^26,43–47^, mainly in the context of memory and cognition^48–50^. We present a systematic empirical study of network interactions among all physiologically relevant cortical rhythms, and discover distinct classes of coupling forms that coexist during a given physiologic state and reorganize with transitions across physiologic states.

We demonstrate the presence of dynamic networks of interactions among brain rhythms. We show that physiologic states can not be fully described by focusing only on individual brain rhythms and on certain pairwise interactions, and that continuous coordination among all brain rhythms as a network underlies physiologic function. Our analyses indicate that the micro-architecture of brain-wave modulation at short timescales (traditionally regarded as noisy fluctuations) carries important information about the nature of brain rhythm interactions. We discover that the interaction between each pair of brain rhythms is characterized by a specific profile, and that collectively, these interactions are represented by an ensemble of profiles (a unique alphabet) that is consistent for all healthy subjects at a given physiologic state. This ensemble of profiles captures the dynamics of reciprocal modulation between different brain rhythms, and demonstrates a transient nature of brain-wave interactions within a given physiologic state. Moreover, we find that brain-wave interaction patterns fall into three distinct classes and change with transition from one physiologic state to another, leading to a hierarchical reorganization of the entire ensemble of interaction profiles. This demonstrates a strong association between the network of coordinated brain-rhythm communications and physiologic states.

In the context of sleep, our findings show that in addition to the traditional framework of defining sleep stages and sleep disorders through markers of dominant cortical rhythms^1,10,24,31^, the specific functional form of coupling and network interactions among both dominant and nondominant cortical rhythms are an essential hallmark of sleep regulation. The reported here distinct classes of brain-wave interaction profiles redefine sleep through a previously unrecognized alphabet of cortical rhythms interactions, and open new perspectives on the regulatory mechanisms of brain dynamics during sleep, with implications for novel biomarkers of sleep and neurological disorders^51,52^. While our findings relate to dynamic network interactions among rhythms derived from the same cortical location (EEG channel), extension of this work will lead to investigations of amplitude correlations and phase relationships between brain waves across cortical locations. The discovery that pairs of brain rhythms exhibit very different functional forms of coupling (an entire alphabet of interaction profiles), which coexist during a given physiologic state and change with transition across states, indicates a previously unrecognized high complexity in the temporal organization of cortical rhythms. Such integrated picture of the interactions among all brain waves as a network is essential to guide future research on modeling the complex interactions of neurons and neuronal populations, and the mechanisms that lead to diverse cortical rhythms with synchronous modulations and different forms of coupling. Our findings will be instrumental in future works using models to bridge spatial scales, from the oscillatory activity at the neuronal level during specific physiological states to functional coupling of integrated brain rhythms at the system level.

Our findings demonstrate that networks of brain-rhythms interactions have a previously unrecognized role as a signature of physiologic state. Currently, there is no mechanistic framework across spatial length scales connecting functional relations between single cell activity at the microscopic level with emerging coupling of integrated brain waves at the macroscopic system level. Our finding of coexisting forms of coupling among cortical rhythms may motivate single cell studies^53^ to probe the origin and mechanisms leading to brain-wave coordination, and open new research directions at the microscopic level to explore the role of heterogeneous neuronal populations in generating distinct classes of brain wave network interactions and associated physiologic functions^54,55^.

In the broader context of complex systems with self-organization and in the framework of Network Physiology^56–58^, our findings raise new questions related to how multi-component coordination among different dynamical processes generates coherent emergent behavior at the integrated level^59–62^. In addition, the uncovered robust alphabet of brain-wave interaction patterns has the potential for broad applications, as one can extend these investigations to other physiologic states (e.g., maturation and age, rest and excise, circadian and ultradian rhythms, stress and vigilance, etc), as well as to various pathological perturbations, psychological, and sleep disorders, where comparative quantification of network maps representative of brain-rhythm communications can lead to new biomarkers of diagnosis and prognosis.

## Methods

### Subjects

Continuous synchronized multi-channel EEG recordings are obtained from 34 healthy young subjects (17 female, 17 male, age between 20 and 40 yrs, average age 29 yrs) during night-time sleep (average record duration 7.6 h) in a sleep laboratory^27^. We utilize pre-existing de-identified multi-channel EEG recordings and sleep-stage annotations from the EU SIESTA database. All participants provided written informed consent. Data use and protocol were approved by the Boston University Charles River Campus Institutional Review Board (IRB protocol number 3380X). Data can be obtained upon request through the advisory board of the SIESTA Group (www.thesiestagroup.com).

For each subject, the recording consists of an electroencephalogram (EEG) using six leads with sampling frequency of 256 Hz. We focus on physiological dynamics during sleep as sleep stages are well-defined physiological states, and external influences due to physical activity or sensory inputs are reduced. Sleep stages are scored in 30 s epochs by sleep lab technicians based on standard criteria^24,28^. Four basic sleep stages are identified: deep sleep (DS), light sleep (LS), rapid eye movement sleep (REM), and wake/arousals (W). Detailed sleep-stage statistics for all subjects are provided in Table 1 in Supplementary Material. A sleep episode is defined as one continuous sleep-stage duration that spans multiple 30 s epochs. While there is some intersubject variability, the sleep-stage statistics are similar across all subjects as indicated by the small standard derivations listed in Table 1 in Supplementary Material. This intersubject variability is typical and does not affect the outcome of our analyses and the reported findings—the brain-wave interaction profiles obtained for individual subjects are consistent with the group average profiles for each pair of brain waves and all sleep stages as also shown by the results of Wilcoxon Signed-rank tests (see “Methods” subsection Distribution profiles of cross-correlation values).

### Brain rhythms relative spectral power

To probe the dynamical interaction among brain waves that are characterized by different EEG frequencies, we apply Fourier band-pass filter to EEG recordings from all six channels. The spectral power in five physiologically relevant frequency bands is extracted from raw EEG signals in consecutive moving windows of 2 s with 1 s overlap: *δ*-wave (0.5–3.5 Hz), *θ*-wave (4–7.5 Hz), *α*-wave (8–11.5 Hz), *σ*-wave (12–15.5 Hz), and *β*-wave (16–19.5 Hz). These are physiologically relevant frequency bands that are widely used in human EEG studies and sleep research. Previous studies have shown that these frequency bands relate to specific physiologic functions (sleep, wake, sleep-stage identification, memory consolidation, attention, arousal activation, etc.), and change under pathologic perturbations^10,27,51^. We note that we purposefully created frequency gaps of 0.5 Hz to better separate the bands. However, our results do not change if we take the original definitions of the bands: *δ* (0–4 Hz), *θ* (4–8 Hz), *α* (8–12 Hz), *σ* (12–16 Hz), *β* (16–20 Hz).

To identify the dynamical evolution and synchronous amplitude modulation of brain rhythms at short timescales, we calculate the relative spectral power in each frequency band with a 1 s resolution. The normalized relative spectral power is obtained as the ratio between the spectral power in the specific frequency band and the total spectral power of all five bands (blue solid lines in Fig. 1a and in Supplementary Fig. 1). Thus, the obtained normalized spectral power represents the relative contribution of each brain rhythm to the total brain activity and allows to quantify reciprocal modulations in the amplitude of different brain waves. To capture the quasi-steady-state behavior of different brain rhythms during different sleep stages, and trends with transitions across sleep stages, we perform a smoothing procedure to the relative spectral power with 14 s moving window and step of 1 s (red solid lines in Fig. 1a and in Supplementary Fig. 1).

The aim of our study is to test the hypothesis of brain-wave interactions mediated through synchronous amplitude modulation of their spectral power bands within the quasi-steady-state behavior of the total EEG power during each 30 s epoch of a given sleep stage. For this, we need to capture the dynamics within 30 s epochs, thus our choice of a smoothing window of approximately half of the 30 s epoch. We note that a too short window of just a few data points does not have the desired smoothing effect to reveal synchronous modulation in the spectral power time series of different brain rhythms at the 1 s resolution of our spectral power analysis. On the other hand, a smoothing window that is too long (longer than half the sleep-stage scoring epoch size of 30 s) will carry information from previous and following sleep epochs, and thus, influences the information derived from our analysis for the considered epoch. Repeating the analysis for moving average window sizes of 10 s and 20 s, we obtained consistent results for all brain-wave interaction profiles (Supplementary Fig. 5).

Since the EEG amplitude heavily impacts the oscillations across all frequency bands, applying our analyses to the absolute power of each frequency band results in strong positive correlations for almost all 30 s windows throughout the entire data set for all pairs of brain waves, and does not lead to differentiation of brain-wave interactions between sleep stages. The reason is that modulations in EEG amplitude (due to intrinsic physiologic regulation or external factors such as movement artifacts and changes in scalp connectivity) lead to global change in the entire power spectrum, and thus to positive cross-correlations among all frequency bands. Repeating the analyses for the absolute spectral power bands we find strong positive cross-correlations with similar profiles for all pairs of brain rhythms across all sleep stages (Supplementary Fig. 4). Considering normalized spectral power for the different brain waves reduces these confounding factors preserving the relative contribution of each brain wave to the total spectral power, and reveals physiologically relevant interaction profiles that change in relation to the different contributions of distinct brain rhythms during different physiologic states (sleep stages) (Fig. 3).

### Cross-correlations between brain rhythms

To probe the dynamical interaction between different brain waves (EEG frequency bands) embedded in short time scale continuous brain-wave fluctuations, we cross-correlate the relative spectral power for each pair of brain waves. Two signals of relative spectral power, {*x*} and {*y*}, each of length *N*, are divided into segments of length *L* and moving step of *M*. We choose *L* = 30 s and *M* = 30 s, which corresponds to the 30-s time resolution of conventional sleep-stage-scoring epochs. Thus, *N*_*L*_ = [(*N* − *L*)/*M*] + 1 is the number of segments where we calculate cross-correlations. Next, we calculate the bivariate equal-time Pearson’s cross-correlation of {*x*} and {*y*} within each segment *ν* = 1,…,*N*_*L*_ as defined by $C_{xy}^\nu (0) = \frac{1}{L} \sum_{i = 1}^L {x_{i + (\nu - 1)M}^\nu y_{i + (\nu - 1)M}^\nu }$. The cross-correlation values range from *C* = −1 (strongest negative cross-correlation) to *C* = 1 (strongest positive cross-correlation). Note, that for two uncorrelated signals *C* = 0. All five brain waves are tested pairwise, leading to ten cross-correlation time series, as shown in Supplementary Fig. 2. This procedure results in an estimation of *N*_*L*_ cross-correlation values *C* for each pair of brain waves. To test the robustness of the the cross-correlation analysis for different timescales, we repeat our procedure for two additional segment lengths *L* = 60 and *L* = 120, and we obtain very similar results. To quantify the dynamics of synchronous amplitude modulation for a specific pair of brain waves during a particular physiologic state, we calculate the distribution of the cross-correlation time series values obtained for all episodes of a given sleep stage over the entire night recording, pooled from all subjects.

### Distribution profiles of cross-correlation values

To quantify coordinated modulation of brain waves, we obtain the distribution of the cross-correlation values *C* for each pair of brain waves during a given physiologic state. Distributions of cross-correlation values for each sleep stage are obtained in the range from *C* = −1 to *C* = 1 using linear bins with size 0.05. To outline the distribution profile, a 4-point moving average is performed (black solid lines in Fig. 1b, c). To confirm that the distribution profile of cross-correlation between each pair of brain waves is robust, we plot the distribution profiles for all subjects together (Supplementary Fig. 3). Further, we pooled together the cross-correlation distribution values of individual subjects to quantify the group average behavior for all pairs of brain waves at a given physiologic state (Fig. 2) and across physiologic states (Fig. 3).

### Statistics and reproducibility

The statistical significance of our results for the brain-wave cross-correlation profiles (Fig. 3) is confirmed by the distribution of *p*-values obtained for the Pearson cross-correlation coefficients for all 30 s windows from all subjects. *P*-values pooled from all subjects after shuffling the spectral power signals in each frequency bands follow a uniform distribution with >96% of the *p*-values above *p* = 0.05, thus confirming the null hypothesis that the analyzed data are random samples (Supplementary Fig. 7a). In contrast, our analysis of real data shows that >80% of the Pearson cross-correlation values pooled from all 30 s segments in all subjects are statistically significant with *p*-values < 0.05 (Supplementary Fig. 7b).

To demonstrate the robustness and consistency of the reported brain-wave interaction profiles across subjects shown in Supplementary Fig. 3, we performed Wilcoxon Signed-rank tests comparing the distribution curves of individual subjects with the group averaged profile. Our null hypothesis (*H* = 0, *p* ≥ 0.05) is that two distribution curves are not different from each other. The test hypothesis (*H* = 1, *p* < 0.05) is that the two curves are different in their functional form. The test results show that the majority of the individual subjects profiles (>94%) are consistent (*p* ≥ 0.05) with the group averaged profile. This indicates that on average not more than two subjects have distribution curves that deviate from the group averaged brain-wave interaction profile for any given pair of brain waves in any sleep stage. Such consistency of the Wilcoxon test results for individual subjects in our database that are recorded in different sleep laboratories shows that inter-scorer variability for sleep staging does not affect our findings.

Under identical procedure of data analysis, we find a diversity in the functional forms of coupling for different pairs of brain waves and four distinct classes of interaction profiles (Fig. 3). This indicates differences in the fine temporal characteristics of spectral power and in the synchronous amplitude modulations for the brain rhythms. An artifact of the methodology of analysis would alternatively show no differences in the coupling forms for the different pairs of brain waves (for example, see Supplementary Fig. 4).

### Degree of cross-correlation between brain rhythms

To further characterize the communication between a given pair of brain rhythms, we define the degree of cross-correlation as the number of episodes with significant cross-correlation values between the two brain waves. We choose a threshold value of *C*_0_ = 0.5, and significant positive cross-correlation is defined as *C* > 0.5, whereas significant anti-correlation corresponds to *C* < −0.5. For a given sleep stage, the degree of cross-correlation is calculated as the fraction of time when significant positive or negative cross-correlation is observed during all episodes of the sleep stage. Thus, for a given pair of brain rhythms the degree of positive correlation is represented by the area under the cross-correlation distribution curve with *C* > 0.5, whereas the degree of anti-correlation corresponds to the area under the distribution curve with *C* < −0.5 (Fig. 4). Choosing the cross-correlation threshold value of *C*_0_ = 0.5 as significance level of coupling among brain rhythms is based on a surrogate analysis, where normalized and smoothed spectral power signals for the frequency bands corresponding to the different brain rhythms are shuffled before performing Pearson’s cross-correlation analysis in 30-s windows. Interaction profiles representing distributions of cross-correlation values obtained from these surrogate data are centered around *C* = 0 and decay rapidly to zero at approximately *C* = ±0.5 (Supplementary Fig. 6a). This surrogate test shows that a degree of positive cross-correlation with *C* > 0.5 and anti-correlation with *C* < −0.5 does not result from a random process but represents relevant physiological interactions. As expected, the highest probability for cross-correlation values obtained from randomly shuffled surrogate data is at *C* ≈ 0, and correspondingly the group average degree of cross-correlation for each surrogate pair of brain rhythms is close to zero (Supplementary Fig. 6b).

We obtain the degree of cross-correlation between all brain rhythms for a typical subject (Fig. 4a) and for the entire group (Fig. 4b). The height of colored (shaded) bars in the upper half of the panels in Fig. 4 represent the degree of positive cross-correlation, while the yellow bars in the lower half of the panels corresponds to the degree of negative cross-correlation. The surrogate test on shuffled data (Supplementary Figs. 6 and 7) confirms the validity of our choice of *C*_0_ = 0.5 as the significance threshold for the degree of cross-correlation among brain rhythms. To further confirm the stability of our results in respect to the choice of the threshold *C*_0_—namely, the robustness of our finding of specific classes of interaction among the brain waves and pronounced sleep-stage dependence of these interactions (Figs. 3–5) we repeat the analysis for different cross-correlation threshold values: *C*_0_ = 0.3, 0.4, 0.6, 0.7. We find that the degree of cross-correlation associated with each pair of brain rhythms does not change significantly for these threshold values of *C*_0_, and exhibits the same stratification pattern when comparing different classes of interactions for a given sleep stage and across sleep stages (Supplementary Fig. 8).

We repeat the calculation of the degree of cross-correlation for all physiologic states as well as for all EEG signals derived from different EEG channels (Fp1, Fp2, C3, C4, O1, O2) and obtain consistent results across cortical areas (Supplementary Fig. 9).

We performed inferential statistics^29,30^ based on the empirical results of the 34 subjects in our database. Inferential statistics address the question on what is the confidence level for the degree of brain wave cross-correlations provided additional subjects are randomly drawn from the same population and analyzed. Inferential statistics present a projection of the boundaries of expected results form the analysis of new subjects based on the limited statistics of the 34 healthy subjects across four sleep stages considered in our database. From the values of the degree of positive and negative cross-correlations from all individual subjects (Fig. 4b; Supplementary Fig. 3), the group mean of the degree of positive and negative cross-correlations (Fig. 4c; Supplementary Fig. 9) and their covariance matrix elements, we obtain the 95% confidence level using bivariant Gaussian distribution. The confidence area is marked as shaded ellipse for each pair of brain waves and all sleep stages in the panels of Supplementary Figs. 12 and 13. The inferential statistics show high consistency for expected results for new subjects drawn from the same population and physiological conditions with the findings obtained from the current database of 34 subjects—with more than 95% probability, data points representing the degree of coupling between cortical rhythms from additional subjects would fall within the area marked by the ellipse in each panel of Supplementary Figs. 12 and 13.

### Dynamic network interactions among brain rhythms

The degree of cross-correlation provides a simple way to characterize the alphabet of brain wave interactions as it reduces each distribution profile to two values, while preserving the important features (e.g., skewness of distribution) of brain-wave communications. To dissect the complex interactions among multiple brain rhythms, we construct positively- and anti-correlated networks where network nodes represent five different brain rhythms, and links represent the degree of cross-correlation between corresponding brain rhythms (Fig. 5). The network link strength corresponds to the group averaged degree of cross-correlation obtained for different pairs of brain rhythms, and is scaled with the line thickness and color (thicker and darker lines represent stronger links). Coexistence of both positively- and anti-correlated networks for each physiologic state demonstrates a transient nature of brain wave communication as it can switch on/off at different times within the same physiologic state. The emergence of different sub-clusters in both networks indicates reorganization in network communications between certain brain rhythms (clustered nodes) and their role in physiologic regulation.

### Reporting summary

Further information on research design is available in the Nature Research Reporting Summary linked to this article.

## Supplementary information

Supplementary Information

Reporting Summary

## Data Availability

The data we used in this work are pre-existing de-identified multi-channel EEG recordings and sleep-stage annotations from the EU SIESTA database. The detailed protocol of the SIESTA database can be found in Klösch et al.^27^. All participants provided written informed consent. Data use and protocol were approved by the Boston University Charles River Campus Institutional Review Board (IRB protocol number 3380X). Data can be obtained upon request through the advisory board of the SIESTA Group (www.thesiestagroup.com).

## Code availability

For data analyses standard MATLAB subroutines (spectral power, window averaging, cross-correlation analysis) are used in particular order as explained in the Methods section.
