# Author Correction: Dynamic network interactions among distinct brain rhythms as a hallmark of physiologic state and function

**DOI:** 10.1038/s42003-020-0998-x

**Published:** 2020-05-21

**Authors:** Aijing Lin, Kang K. L. Liu, Ronny P. Bartsch, Plamen Ch. Ivanov

**Affiliations:** 1 Department of Mathematics, School of Science, Beijing Jiaotong University, Beijing, 100044 China; 2 Keck Laboratory for Network Physiology, Department of Physics, Boston University, Boston, MA 02215 USA; 3 Department of Neurology, Beth Israel Deaconess Medical Center, Harvard Medical School, Boston, MA 02115 USA; 4 Department of Physics, Bar-Ilan University, Ramat Gan, 5290002 Israel; 5 Division of Sleep Medicine, Brigham and Women’s Hospital, Harvard Medical School, Boston, MA 02115 USA; 6 Institute of Solid State Physics, Bulgarian Academy of Sciences, Sofia, 1784 Bulgaria

**Keywords:** Sleep, Circadian rhythms and sleep

Correction to: *Communications Biology* 10.1038/s42003-020-0878-4, published online 27 April 2020.

In the original published version of the article references 56, 57, and 58 contained errors. These have been corrected in the HTML and PDF versions of the article.

