# Supplementary material for: Dynamic network interactions among distinct brain rhythms as a hallmark of physiologic state and function
Source: Commun Biol. 2020 Apr 27;3:197. doi: 10.1038/s42003-020-0878-4 (PMC7184753; doi:10.1038/s42003-020-0878-4)
Supplement: Supplementary file 1 — Supplementary Information [file 42003_2020_878_MOESM1_ESM.pdf]

Supplementary Materials

Supplementary Figures

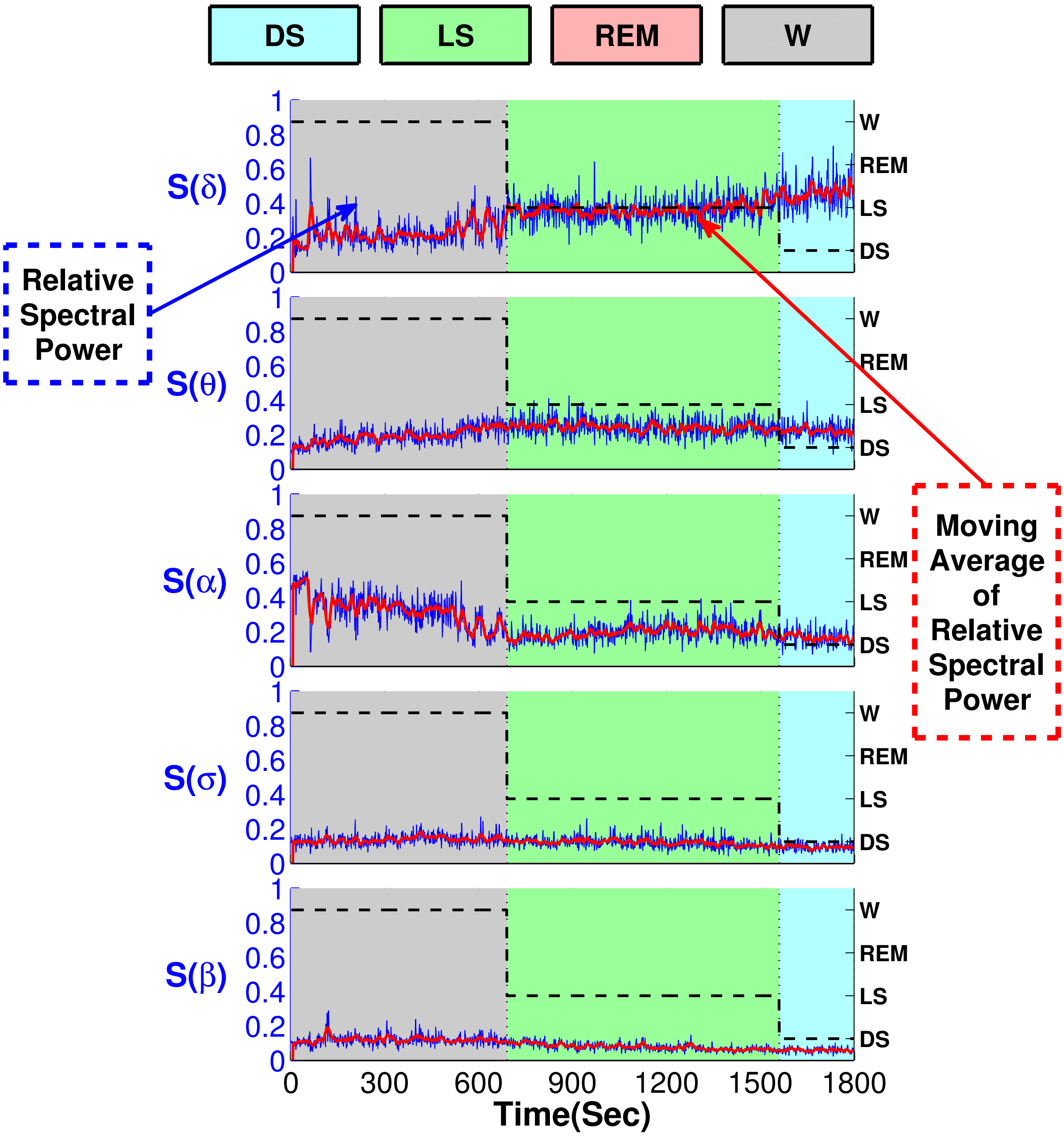

Supplementary Fig. 1

Supplementary Fig. 1: Classical picture representing physiologic states through quasi-steady-state behaviors of dominant brain rhythms. Spectral power of five physiologically relevant brain rhythms (frequency bands: δ [0.5-3.5]Hz, θ [4-7.5] Hz, α [8-11.5] Hz, σ [12-15.5] Hz, β [16-19.5] Hz) derived from brain EEG recordings at the central C3 channel are shown in blue solid lines. Spectral power for each brain rhythm is calculated with time resolution of 1 sec and is normalized by the total spectral power of all five rhythms, i. e., at each time the sum of all five renormalized spectra is one. Red lines represent the average of the spectral power for each brain rhythm obtained for a moving window of 14 sec and step of 1 sec. Black dashed lines represent sleep stages as defined by traditional sleep-stage scoring criteria (24, 27). Shown is a typical 30 min segment of recording at the beginning of night time sleep. As the subject falls asleep from quiet wake (W) and then undergoes a transition from light sleep (LS) to deep sleep (DS), there is a significant change in the spectral power of brain rhythms — δ power significantly increases and becomes dominant during DS, while α rhythm declines. Changes are also observed in the spectral power of other non-dominant brain rhythms (θ, σ and β), although less pronounced. During a given physiologic state, the spectral power of each brain rhythm can be approximated by a quasi-steady-state with superposed continuous noisy fluctuations at short scales (amplitude modulations), the nature of which has not been systematically studied and remains not well understood. Our analyses reveal important physiologically relevant information on interactions among brain waves embedded in these fluctuations.

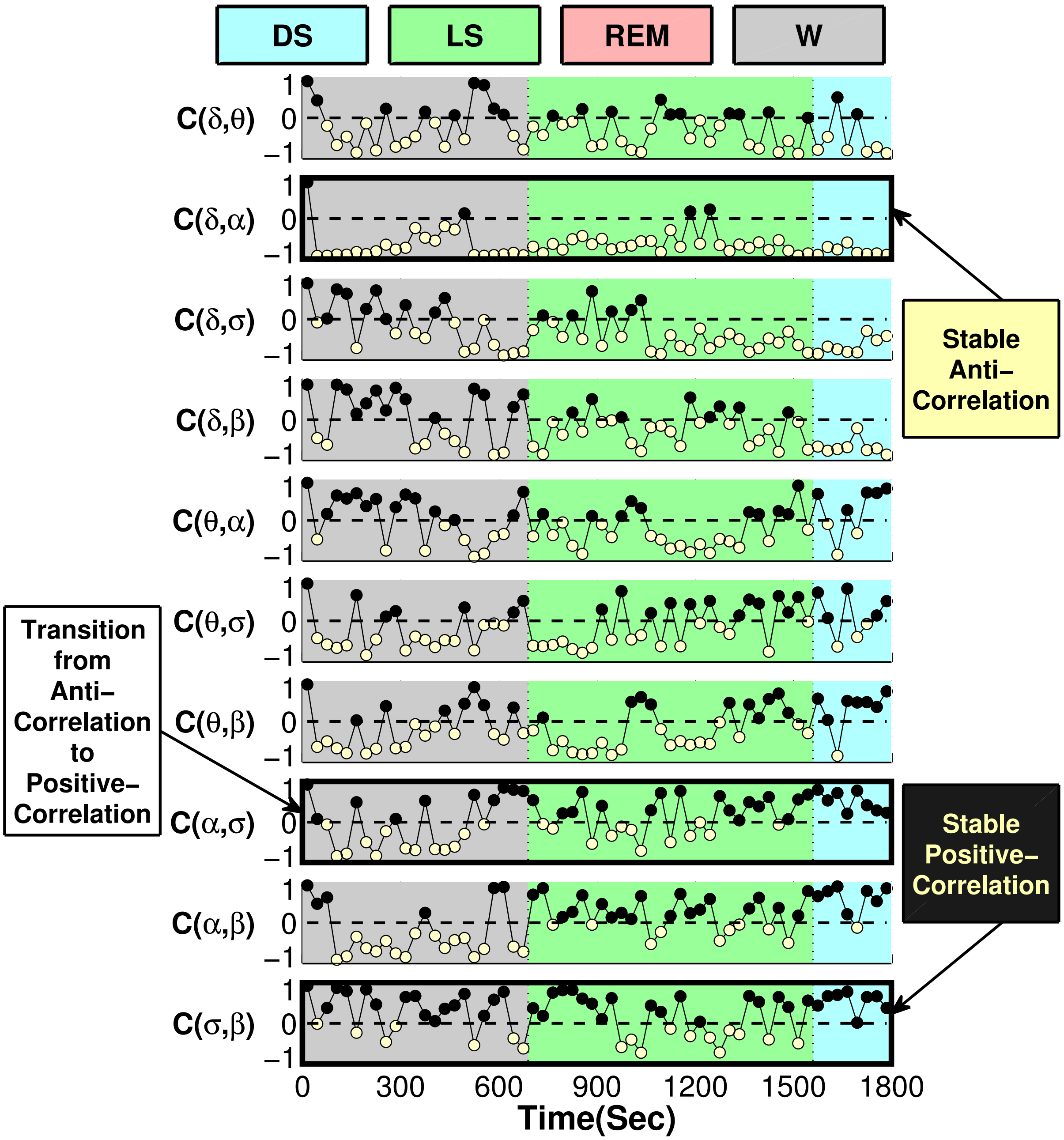

Supplementary Fig. 2

Supplementary Fig. 2: Temporal dynamics of brain wave interactions and transitions across physiologic states. Cross-correlation function without time delay between brain rhythms is calculated in non-overlapping windows of 30 sec. Shown are the cross-correlation functions between all ten pairs of the five brain rhythms during the same time period as in Supplementary Fig. 1. Black (full) symbols represent positive correlation and yellow (open) symbols correspond to anti-correlation. Different pairs of brain rhythms exhibit different type of cross-correlation dynamics, and three distinct classes are observed among all ten pairs of brain rhythms: (i) class of brain waves that exhibit stable anti-correlation during all stages, such as $\delta - \alpha$ interaction; (ii) pairs of brain waves that show stable positive-correlation in all sleep stage, such as $\sigma - \beta$ interaction; (iii) pairs of brain waves that change their interaction from anti-correlation to positive correlation with transition from W to LS and DS (such as $\alpha - \sigma$ and $\alpha - \beta$ interactions). These cross-correlation functions probe and quantify the dynamical interactions between brain rhythms derived from the high-frequency modulation in their amplitudes at much shorter time scales compared to the large-scale steady-state behavior in the classical description of physiologic states shown in Supplementary Fig. 1. These sleep-stage dependent dynamics of brain wave communications indicate that fluctuations in brain wave spectral power at short time scales (Fig. 1 and Supplementary Fig. 1) carry important information about brain rhythm interactions and the underlying mechanism of physiologic regulation.

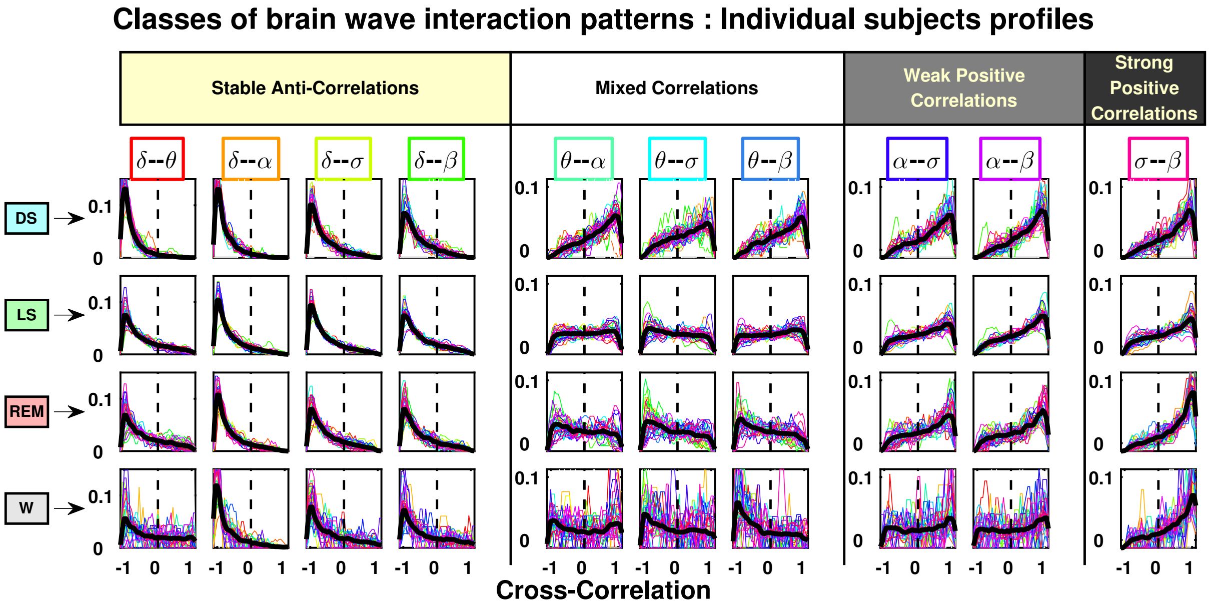

Supplementary Fig. 3

Supplementary Fig. 3: Universal patterns of brain wave interactions across different subjects. Profiles of cross-correlation distributions for each pair of brain waves during different physiologic states (sleep stages) plotted for all 34 subjects in our database (Section Materials and Methods). To compare distributions from individual subjects with pooled data that represent group averaged behavior, all cross-correlation histograms of individual subjects are renormalized by the total number of cross-correlation values (obtained in 30 sec epochs) for each subject, so that the area under each distribution curve equals one. Line colors correspond to different subjects and solid black line represents the pooled distribution from all subjects (as also shown in Fig. 3). For each pair of brain wave interaction during a particular sleep stage (horizontal row) all distributions collapse onto a single curve, indicating that the observed characteristics of brain wave interactions within each sleep stage are universal for all subjects. Remarkably, data collapse of cross-correlation distributions is consistently observed for all pairs of brain waves during all sleep stages, indicating that the alphabet of brain wave communications is a robust signature of physiologic state.

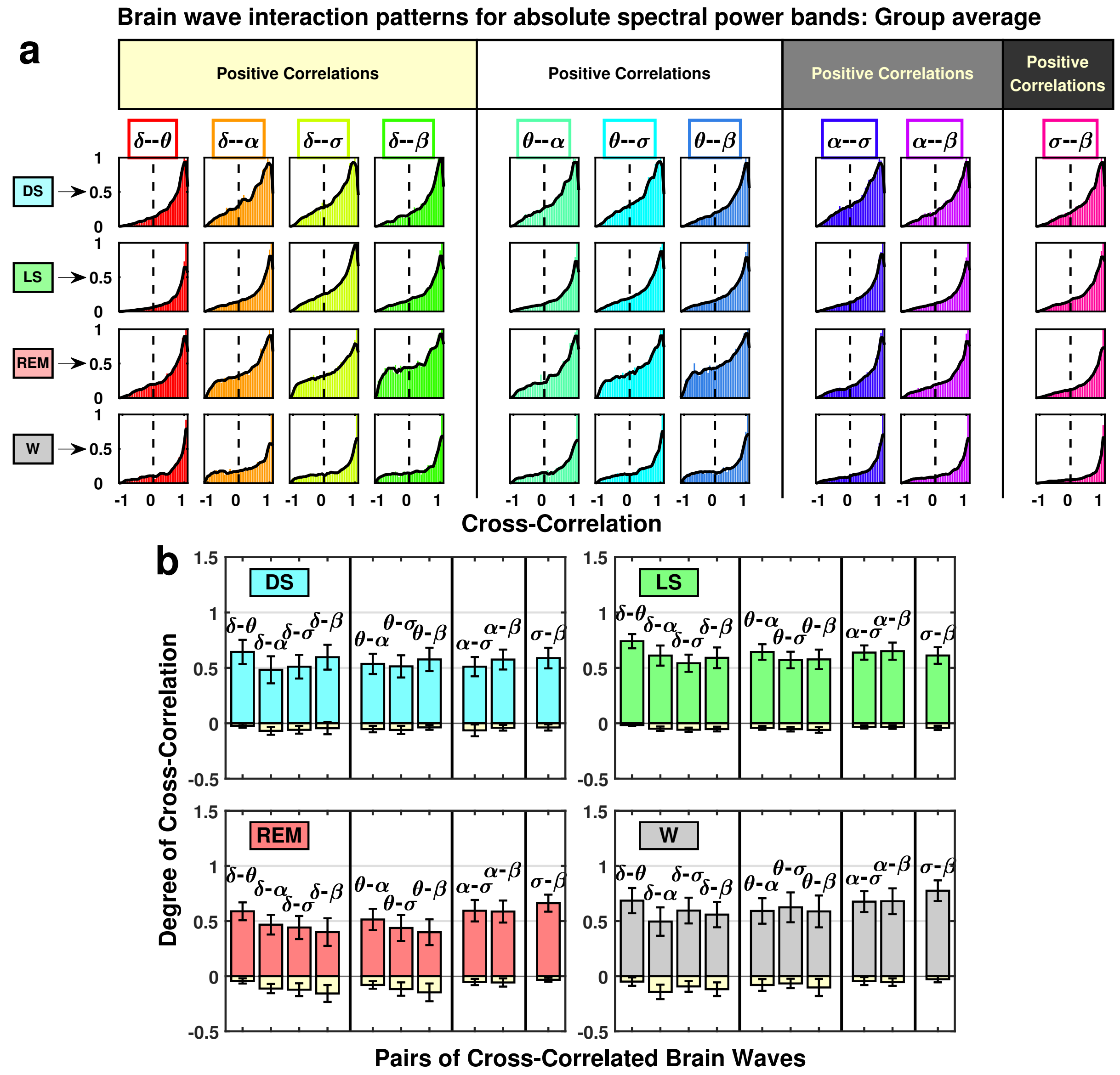

Supplementary Fig. 4

Supplementary Fig. 4: Analysis of brain wave coupling using their absolute spectral power. (a) Group averaged interaction profiles for pairs of brain waves obtained using the absolute spectral power in their corresponding frequency bands (as defined in Section Materials and Methods). Profiles represent rescaled histograms of Pearson cross-correlation coefficients calculated in 30 sec windows pooled from all subjects (Materials and Methods). Due to global

modulations in EEG amplitude and correspondingly in total EEG spectral power in response to changes in intrinsic physiologic regulation and external factors (e.g., movement artifacts and scalp connectivity) all frequency bands are simultaneously affected in the same direction of increasing or decreasing their absolute power, thus leading to similar positive cross-correlation profiles for all pairs of brain waves across all sleep stages. (b) Bars represent the degree of cross-correlation for the pairs of brain waves shown in panel a. The degree of cross-correlation is calculated as the fraction of time when significant positive or negative cross-correlation is observed for a pair of brain waves during all episodes of the sleep stage (Materials and Methods, Fig. 4 in the manuscript). Shown are group averages and standard deviations. Results indicate strong positive cross-correlations for all pairs of brain waves with no differentiation between pairs for a given sleep stage and similar pattern for all sleep stages (no sleep-stage stratification). In contrast, analyses of the normalized spectral power of the frequency bands demonstrate distinct classes of coupling and response of brain wave interactions to changes in physiologic state characterized by pronounced sleep-stage stratification.

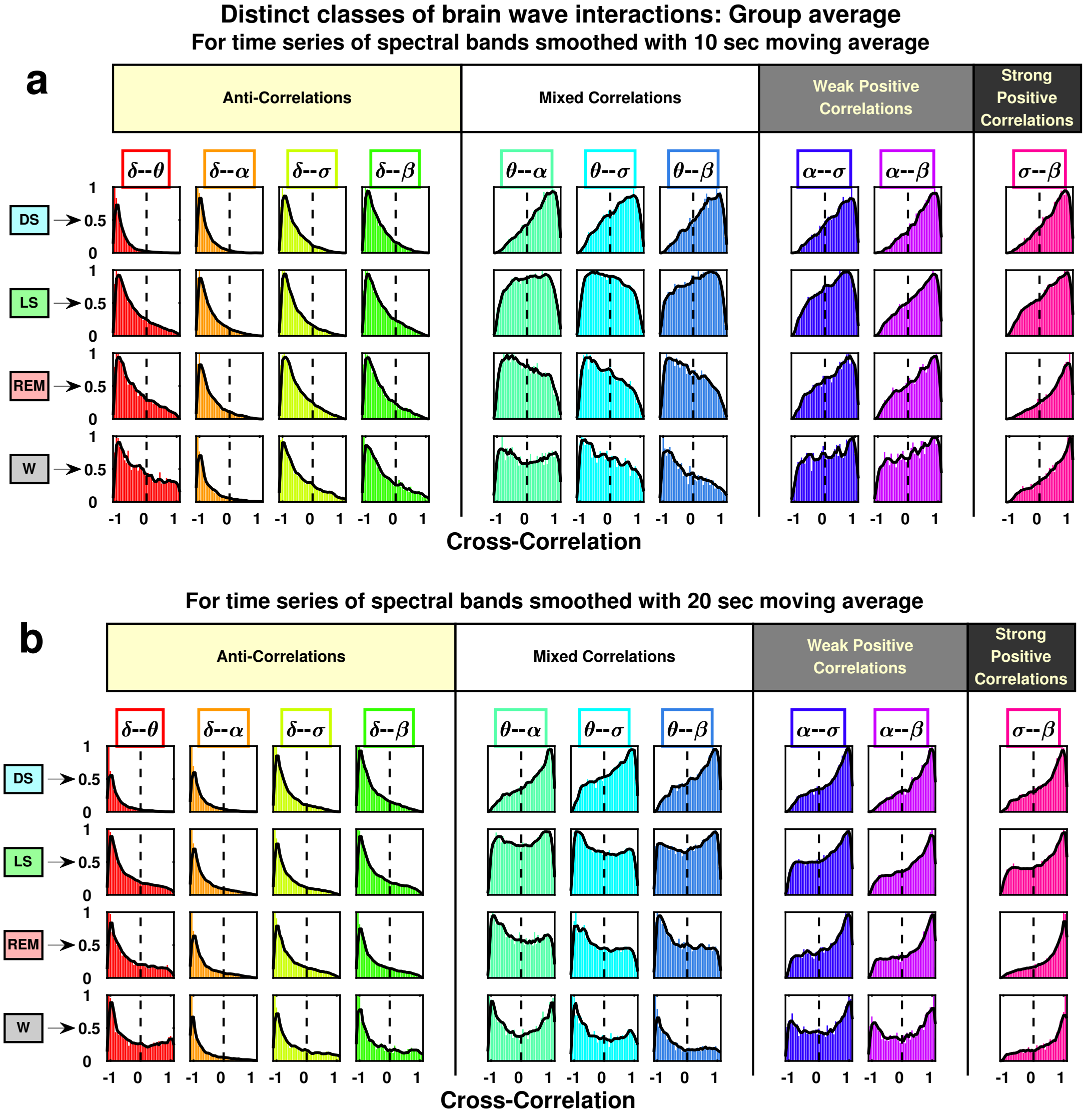

Supplementary Fig. 5

Supplementary Fig. 5: Brain wave interaction profiles obtained for different moving average window sizes applied to smooth the spectral power time series for the different frequency bands. Results are consistent with those obtained for a 14 sec moving average window shown in Fig. 3.

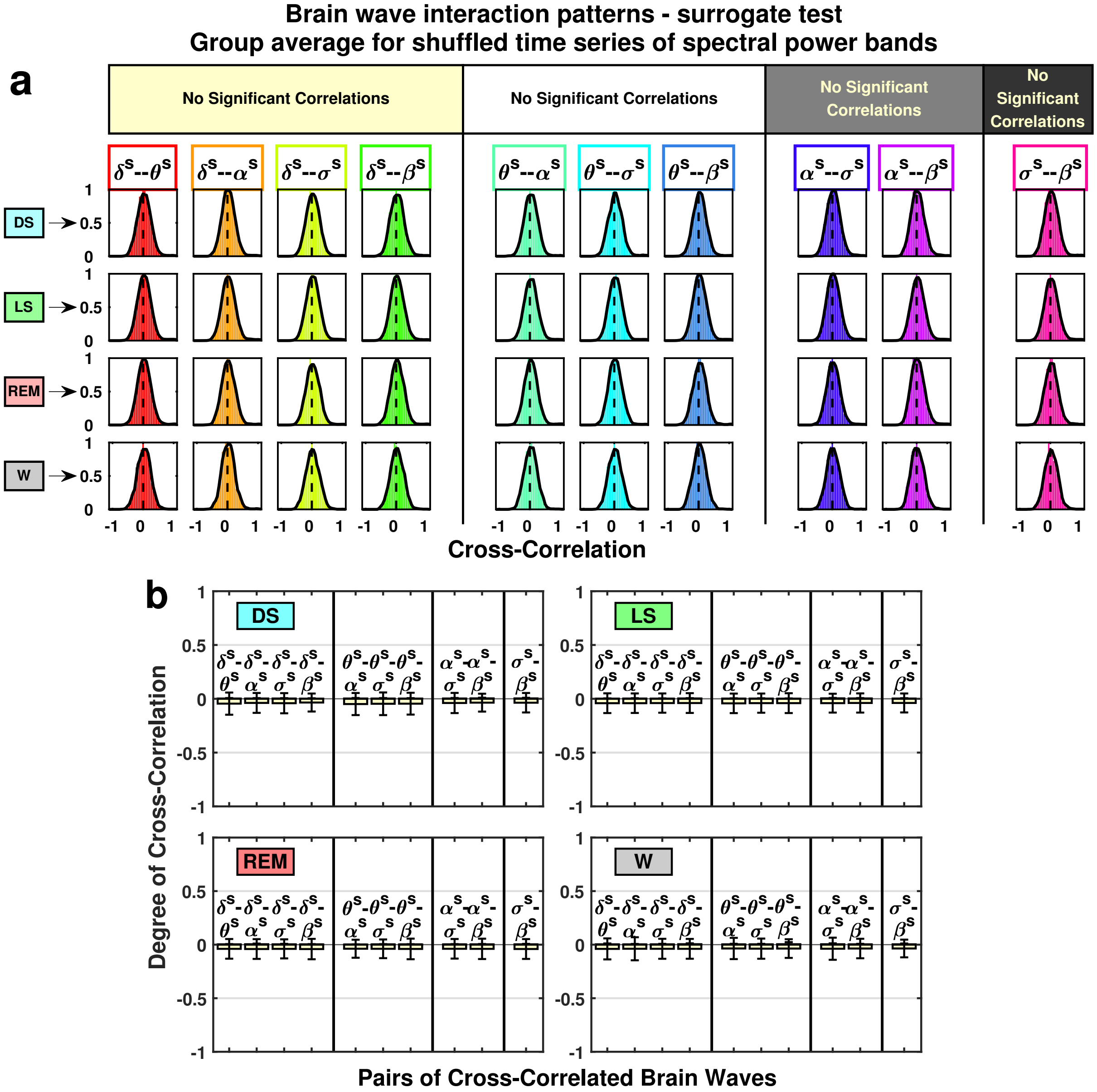

Supplementary Fig. 6

Supplementary Fig. 6: Surrogate test based on shuffled data to define a significance threshold for cross-correlation values. (a) Interaction profiles are obtained starting with normalized spectral power in each frequency band and randomly shuffling the time series for all five bands before pairing them and applying cross-correlation analysis in 30 sec non-overlapping win-

dows. This surrogate test shows the effect of our procedure on random signals derived from the original data to establish a minimum baseline for cross-correlation significance. As expected, cross-correlation values are distributed around $C \approx 0$ with variance due to the finite window size of 30 sec (i.e., 30 data points are used to calculate each Pearson cross-correlation coefficient). The distribution profiles obtained for all surrogate pairs of frequency bands are practically identical for all sleep stages, with probability going to zero at $C = \pm 0.5$ – i.e., a significance cross-correlation threshold for our analyses. The test indicates that the distinct classes of profiles and their sleep-stage stratification obtained from real data (Fig. 3) reflect underlying physiologic interactions among brain rhythms. (b) Group average statistics for the degree of cross-correlation derived from the surrogate test profiles in panel a. Results show a degree of cross-correlation approximately zero beyond the threshold $C = \pm 0.5$.

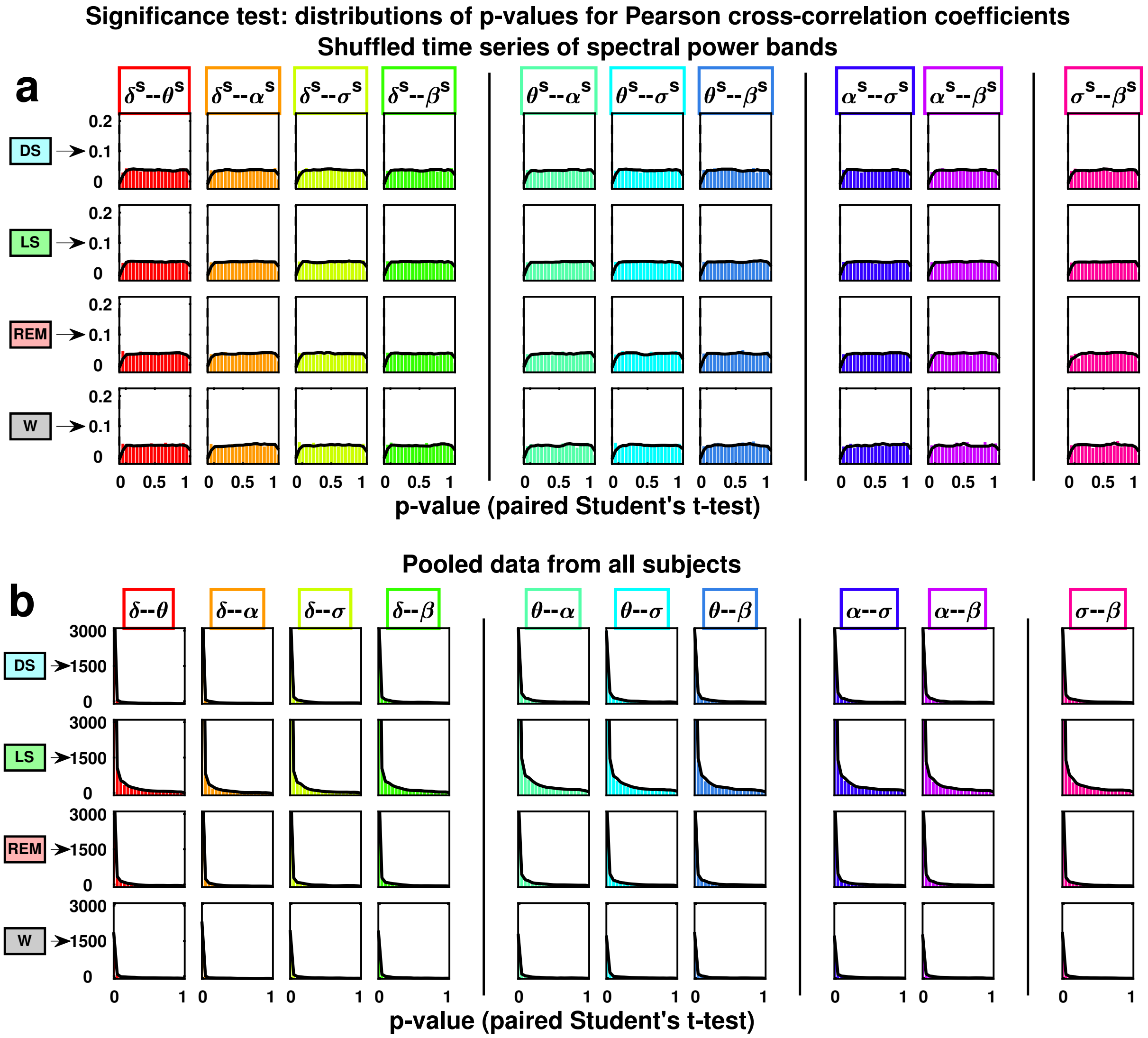

Supplementary Fig. 7

Supplementary Fig. 7: Test for statistical significance of the distinct classes of brain wave interaction profiles. (a) Normalized distributions of p-values for the Pearson cross-correlation coefficients obtained for all 30 sec windows and pooled from all subjects after shuffling the spectral power frequency bands of the paired brain waves (corresponding to the profiles from the surrogate shuffled data test shown in Fig. 6). Uniform distributions with >96% of the p-values above $p = 0.05$ for all pairs of frequency bands and all sleep stages confirm the null hypothesis

that the analyzed data are random. (b) Histograms of the Pearson cross-correlation coefficients p-values obtained from real data and pooled from all 30 sec windows and all subjects. In contrast to panel a, all histograms exhibit peak at $p < 0.05$ rejecting the null hypothesis that the analyzed data are random samples, and thus confirming the statistical significance of the reported distinct classes of brain wave interaction profiles in Fig. 3.

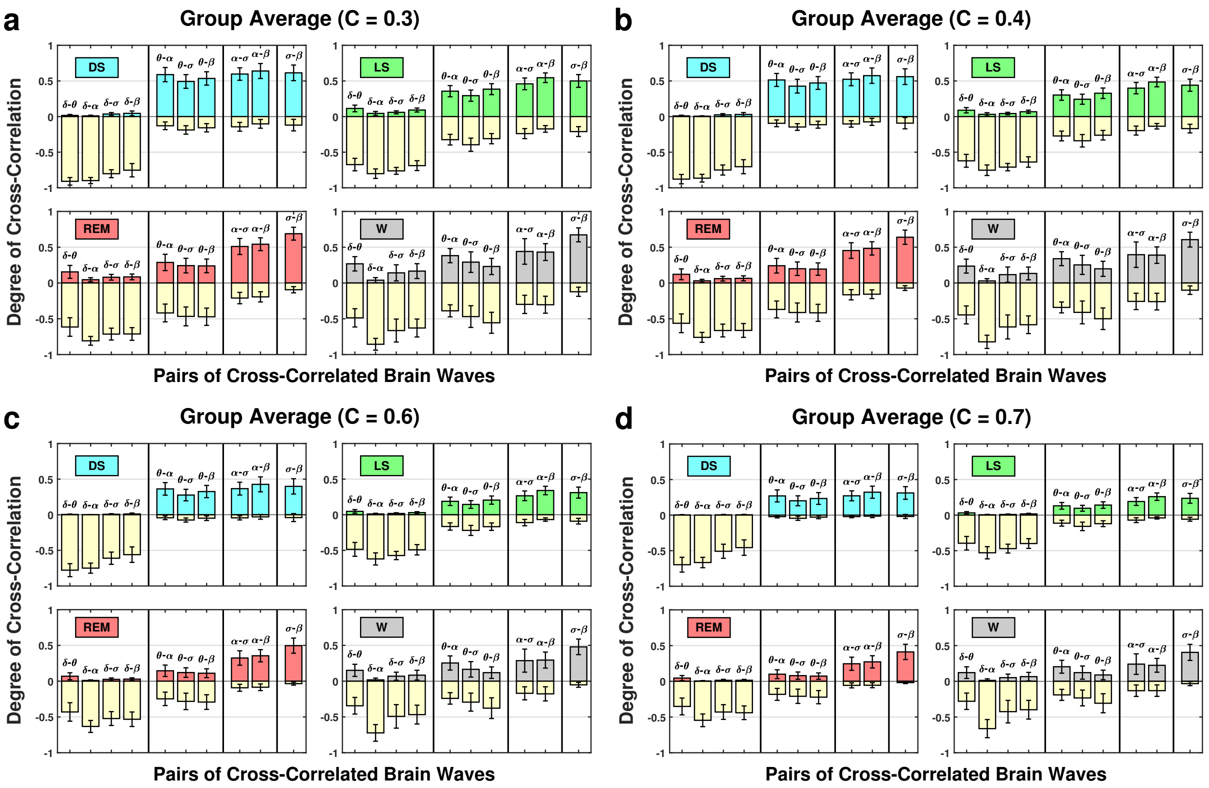

Supplementary Fig. 8

Supplementary Fig. 8: Robust classes of brain wave interactions and stable sleep-stage stratification patterns. Shown are the results for four cross-correlation threshold values (a) $C = 0.3$, (b) $C = 0.4$, (c) $C = 0.6$, and (d) $C = 0.7$ imposed on the profiles in Fig. 3 to quantify the degree of cross-correlation shown in Fig. 4a. For each pair of brain waves, the positive bars represent the degree of positive cross-correlations calculated as the percentage of the interaction profile area above the threshold C, and negative bars represent the degree of anti-correlations calculated as the percentage of the profile area below the threshold $-C$. Error bars represent the group standard deviation. Colors of positive bars correspond to sleep stages, and all negative bars are shown in yellow. The consistency of our results for different cross-correlation threshold values C indicate a robust physiological mechanism underlying the coupling profiles of brain rhythms, their organization in distinct classes and their stratification across sleep stages.

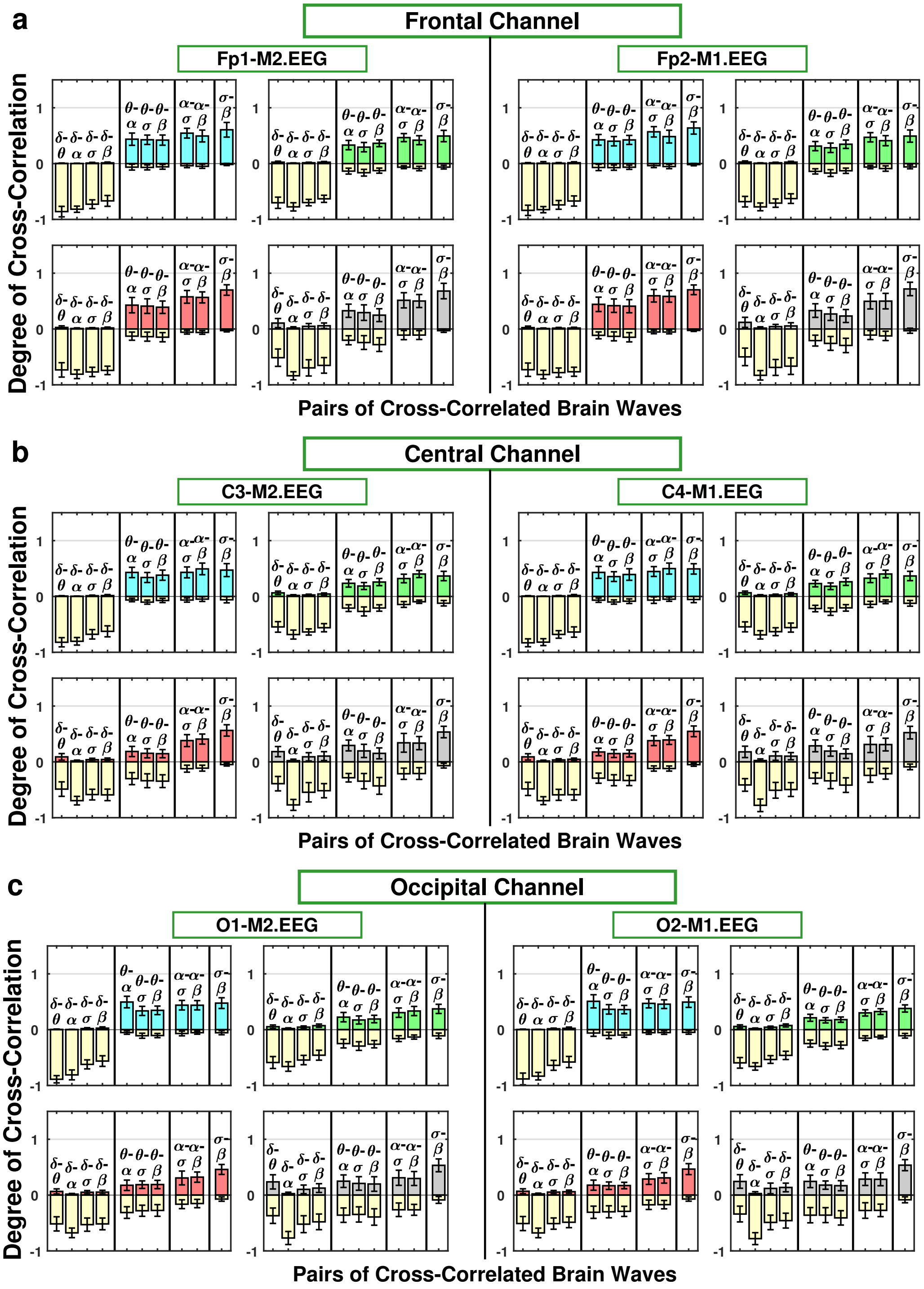

Supplementary Fig. 9

Supplementary Fig. 9: Consistent patterns of brain wave interactions across different brain areas. For each pair of brain waves, positive and negative bars represent the fraction of the recording during a specific physiologic state (sleep stage) with significant positive correlation ($C > 0.5$) and significant anti-correlation ($C < -0.5$), respectively. Three major classes of brain wave interactions are clearly observed across the Frontal (Fp1 and Fp2 channels), Central (C3 and C4) and Occipital (O1 and O2) brain areas: (i) pairs of brain waves that are strongly anti-correlated (large negative bars) for all sleep stages, as shown by the first 4 brain wave pairs in each panel; (ii) pairs of brain waves that are strongly positively-correlated (large positive bars) for all sleep stages, as shown by the last 3 brain wave pairs in each panel; (iii) pairs of brain waves that switch from positively- to anti-correlated interactions with transitions across sleep stages (switch from large positive to large negative bars, as shown by the 3 pairs in the middle of each panel). Error bars represent group standard deviation. Colors of positive bars correspond to sleep stages, and all negative bars are shown in yellow. We observe a strong symmetry in the cross-correlation patterns of brain wave communication between corresponding brain locations in left and right hemisphere. Notably, during DS and LS we find that all brain areas exhibit similar patterns of brain wave interactions. In contrast, during REM and wake, Frontal areas (Fp1 and Fp2) exhibit markedly different patterns from Central (C3 and C4) and Occipital areas (O1 and O2) with tendency towards increased anti-correlations (larger negative bars). Consistent results are observed at all cortical locations for all pairs of cortical rhythms and sleep stages.

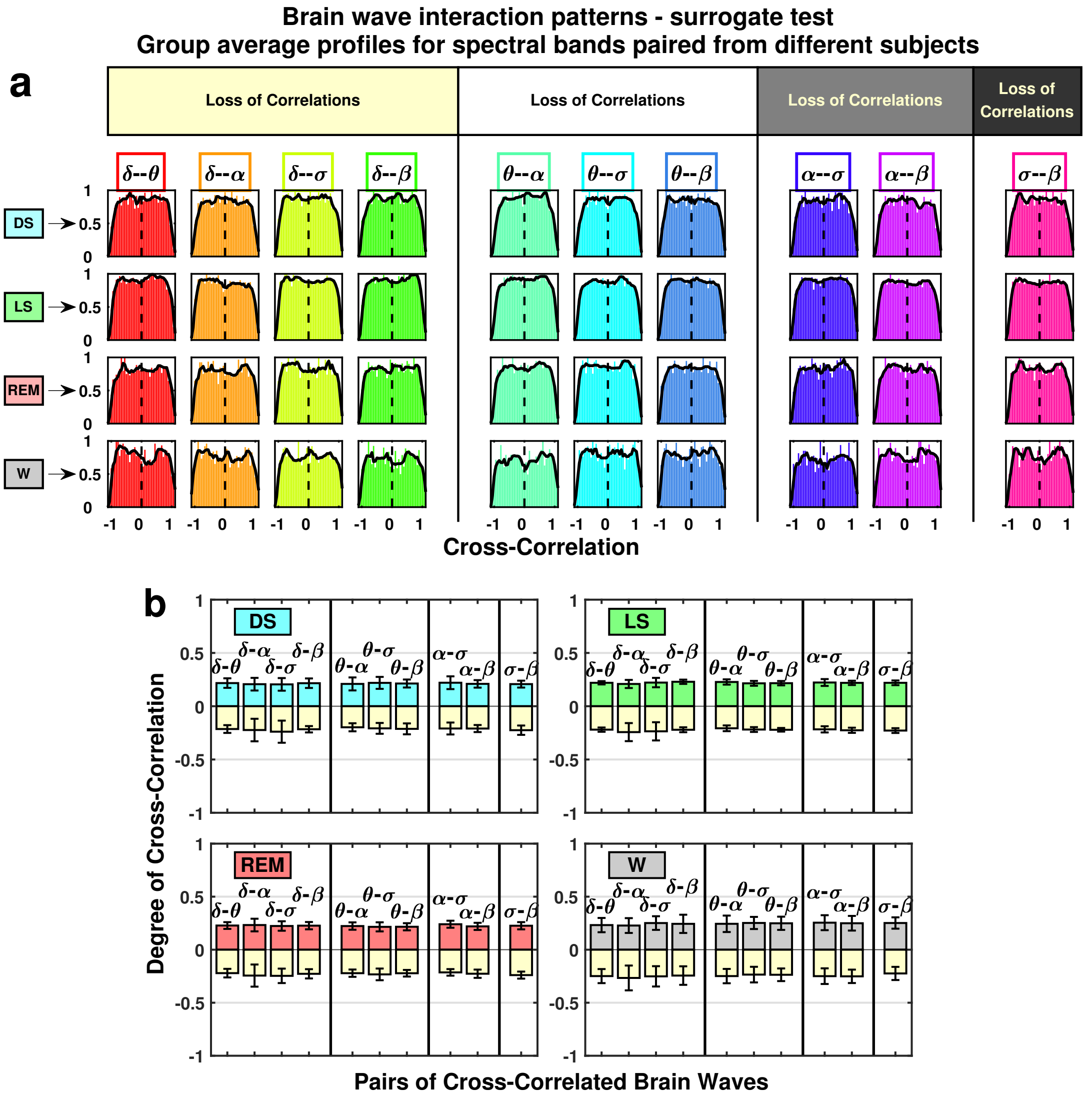

Supplementary Fig. 10

Supplementary Fig. 10: Surrogate test where pairs of brain wave spectral power signals are taken from two different subjects. (a) Distributions of Pearson cross-correlation coefficients obtained by pooling together 34 realizations of surrogate pairs for each combination of brain waves in each sleep stage. Uniform profiles indicate absence of consistent cross-correlations. (b) Degree of cross-correlations derived from the distribution profiles in panel (a) show no differentiation among different surrogate pairs of brain waves and no sleep-stage

stratification. While the original characteristics of the separate brain wave spectral power signals are preserved in this surrogate analysis, the test demonstrates that the reported distinct classes of brain wave interaction profiles (Fig. 3) represent physiological coupling related to synchronous modulation in the amplitudes of different brain waves.

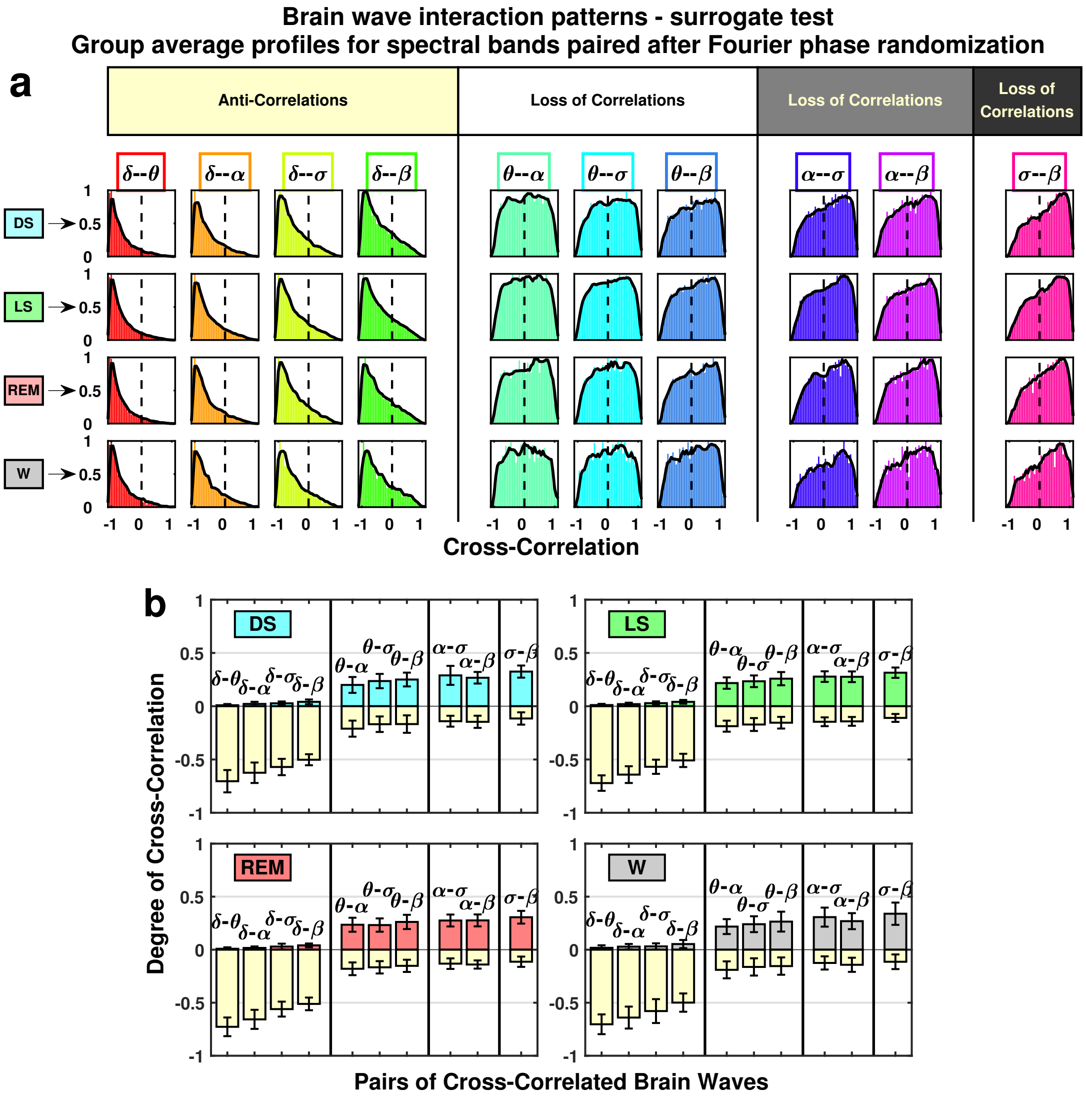

Supplementary Fig. 11

Supplementary Fig. 11: Surrogate test with Fourier Phase randomization of EEG signals. The test preserves the relative spectral power of the frequency bands corresponding to the different brain rhythms but lead to temporal reorganization of their instantaneous amplitudes, thus eliminating synchronous amplitude modulation between brain waves at short time scales. (a) Interaction profiles for all pairs of brain waves during different sleep stages. Profiles of surrogate data change compared to real data shown in Fig. 3. (b) Degree of cross-correlation for Fourier

phase randomized surrogate data shows no sleep-stage stratification, which is in contrast to results from real data (Fig. 4). The surrogate test demonstrates that the reported functional forms of brain wave coupling reflect physiological information related to both reorganization in the spectral power of different brain waves at large time scales as well as in their synchronous modulation at short time scales that occur in response to change in physiologic regulation during different sleep stages.

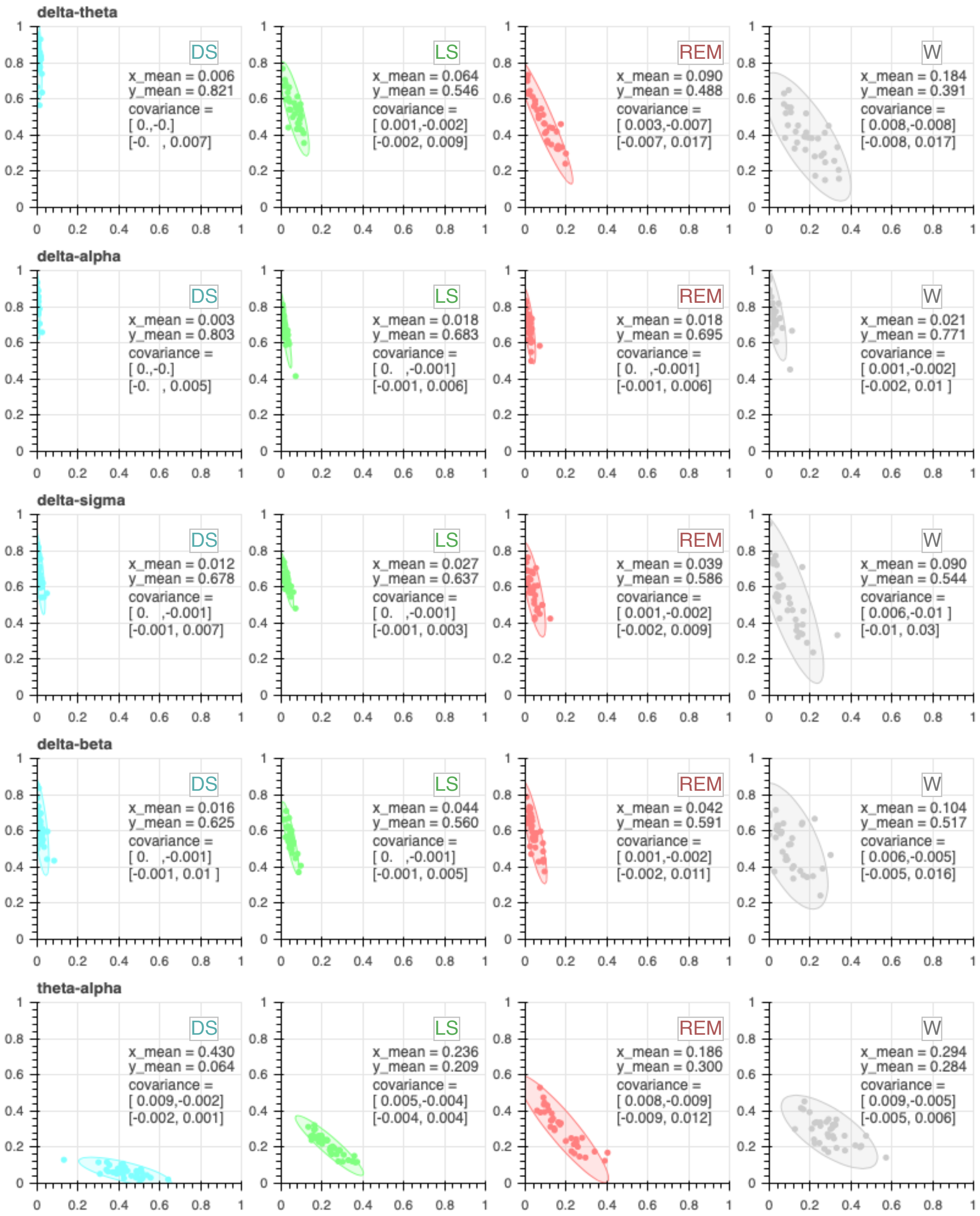
delta-theta
DS
x_mean = 0.006
y_mean = 0.821
covariance =
[0.,-0.]
[-0. , 0.007]
LS
x_mean = 0.064
y_mean = 0.546
covariance =
[0.001,-0.002]
[-0.002, 0.009]
REM
x_mean = 0.090
y_mean = 0.488
covariance =
[0.003,-0.007]
[-0.007, 0.017]
W
x_mean = 0.184
y_mean = 0.391
covariance =
[0.008,-0.008]
[-0.008, 0.017]
delta-alpha
DS
x_mean = 0.003
y_mean = 0.803
covariance =
[0.,-0.]
[-0. , 0.005]
LS
x_mean = 0.018
y_mean = 0.683
covariance =
[0. ,-0.001]
[-0.001, 0.006]
REM
x_mean = 0.018
y_mean = 0.695
covariance =
[0. ,-0.001]
[-0.001, 0.006]
W
x_mean = 0.021
y_mean = 0.771
covariance =
[0.001,-0.002]
[-0.002, 0.01]
delta-sigma
DS
x_mean = 0.012
y_mean = 0.678
covariance =
[0. ,-0.001]
[-0.001, 0.007]
LS
x_mean = 0.027
y_mean = 0.637
covariance =
[0. ,-0.001]
[-0.001, 0.003]
REM
x_mean = 0.039
y_mean = 0.586
covariance =
[0.001,-0.002]
[-0.002, 0.009]
W
x_mean = 0.090
y_mean = 0.544
covariance =
[0.006,-0.01]
[-0.01, 0.03]
delta-beta
DS
x_mean = 0.016
y_mean = 0.625
covariance =
[0. ,-0.001]
[-0.001, 0.01]
LS
x_mean = 0.044
y_mean = 0.560
covariance =
[0. ,-0.001]
[-0.001, 0.005]
REM
x_mean = 0.042
y_mean = 0.591
covariance =
[0.001,-0.002]
[-0.002, 0.011]
W
x_mean = 0.104
y_mean = 0.517
covariance =
[0.006,-0.005]
[-0.005, 0.016]
theta-alpha
DS
x_mean = 0.430
y_mean = 0.064
covariance =
[0.009,-0.002]
[-0.002, 0.001]
LS
x_mean = 0.236
y_mean = 0.209
covariance =
[0.005,-0.004]
[-0.004, 0.004]
REM
x_mean = 0.186
y_mean = 0.300
covariance =
[0.008,-0.009]
[-0.009, 0.012]
W
x_mean = 0.294
y_mean = 0.284
covariance =
[0.009,-0.005]
[-0.005, 0.006]

Supplementary Fig. 12: Inferential statistics on the degree of positive and negative cross-correlation for the coupling of all pairs of brain waves and all sleep stages. Data points correspond to the 34 individual subjects in the analyzed database with degree of positive cross-correlations on the x-axis, and negative cross-correlations on the y-axis. Group means for the positive and negative cross-correlations and the covariance matrix is given in each panel. Panels in separate rows show data from each pair of brain waves across four sleep stages. Shaded ellipse in each plot indicates the 95% confidence interval. Tests show consistency between the obtained results based on the limited database of 34 subjects we analyzed (dots in each panel) and the expected results from a much larger database of the same population.

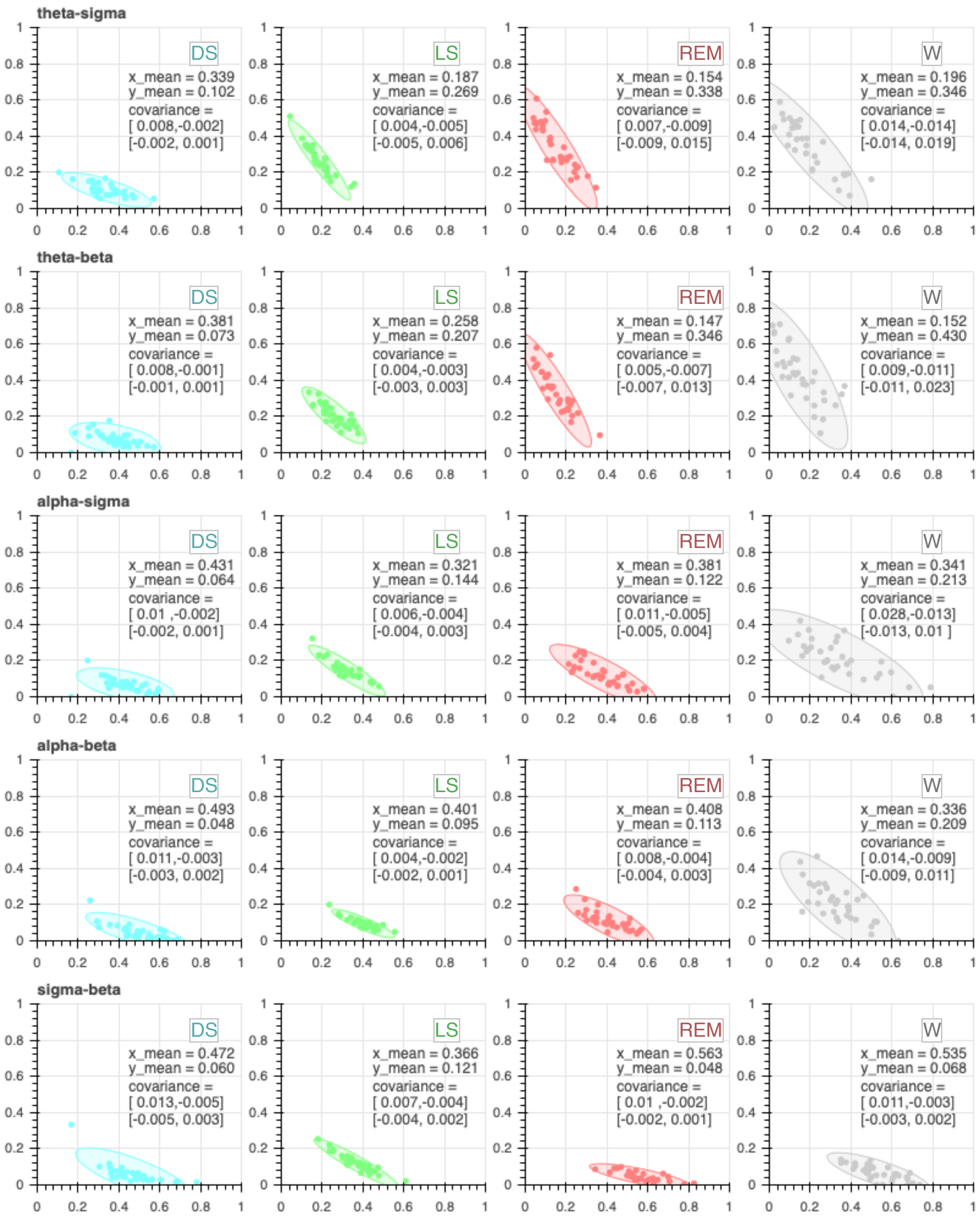

Supplementary Fig. 12

Supplementary Fig. 13: Inferential statistics on the degree of positive and negative cross-correlation for the coupling of all pairs of brain waves and all sleep stages. Data points correspond to the 34 individual subjects in the analyzed database with degree of positive cross-correlations on the x-axis, and negative cross-correlations on the y-axis. Group means for the positive and negative cross-correlations and the covariance matrix is given in each panel. Panels in separate rows show data from each pair of brain waves across four sleep stages. Shaded ellipse in each plot indicates the 95% confidence interval. Tests show consistency between the obtained results based on the limited database of 34 subjects we analyzed (dots in each panel) and the expected results from a much larger database of the same population.

Supplementary Tables

Subjects	Record Length (Hours)	Number of Episodes				Stage Duration (%)			
		DS	LS	REM	W	DS	LS	REM	W
**Subject01**	8	4	13	6	5	13.6	65.0	18.2	3.2
**Subject02**	8	6	14	6	2	17.5	60.3	21.3	0.9
**Subject03**	7.9	6	23	12	8	9.8	58.0	18.6	13.6
**Subject04**	8.1	5	23	9	17	13.3	48.3	19.1	19.3
**Subject05**	8	7	16	7	3	10.3	62.2	25.5	2.0
**Subject06**	7.9	7	22	10	12	20.3	44.2	26.0	9.5
**Subject07**	6.7	7	14	6	4	22.4	44.7	25.0	8.0
**Subject08**	8	4	14	7	8	13.1	48.8	22.0	16.1
**Subject09**	7.8	8	19	10	6	16.4	56.5	22.9	4.2
**Subject10**	8.1	7	19	5	15	14.8	57.2	18.4	9.5
**Subject11**	7.4	4	16	6	7	9.6	65.8	14.0	10.7
**Subject12**	8.2	4	19	6	13	6.6	65.6	15.6	12.3
**Subject13**	8.6	8	18	8	7	14.2	56.8	14.9	14.0
**Subject14**	8.8	5	18	8	12	10.1	59.8	17.2	13.0
**Subject15**	8.2	4	22	8	16	10.6	67.5	18.8	13.1
**Subject16**	7.2	3	19	14	4	23.5	45.8	27.0	3.6
**Subject17**	8.2	13	31	13	7	16.6	60.0	16.6	6.8
**Subject18**	6.5	7	19	9	9	24.5	50.6	18.3	6.6
**Subject19**	8.8	5	26	11	15	19.7	49.4	19.6	11.3
**Subject20**	7.5	6	19	9	10	14.6	56.2	23.2	6.0
**Subject21**	8	6	23	10	15	17.0	55.8	19.3	7.9
**Subject22**	7.7	9	22	10	5	20.3	55.1	20.1	4.5
**Subject23**	8	7	20	6	13	17.5	55.3	22.8	4.4
**Subject24**	8	5	14	7	8	15.4	58.2	21.6	4.8
**Subject25**	8	8	21	8	6	19.4	58.1	20.2	2.3
**Subject26**	7.8	8	18	7	5	26.2	43.9	26.6	3.3
**Subject27**	7.6	11	33	16	20	21.2	52.3	15.8	10.8
**Subject28**	8	8	26	12	8	19.3	55.5	18.3	6.9
**Subject29**	6.9	6	22	11	7	21.2	58.8	15.3	4.7
**Subject30**	8	5	22	10	12	14.6	59.3	20.3	5.7
**Subject31**	8.1	11	22	7	8	22.4	52.9	21.8	2.9
**Subject32**	8	5	17	4	9	17.5	52.8	12.1	17.6
**Subject33**	8	8	29	11	17	12.5	55.3	20.6	11.7
**Subject34**	8	3	18	6	13	19.7	42.1	21.6	16.5
**Mean**	7.8	6.5	20.3	8.7	9.6	16.6	55.2	20.0	8.5
**Std**	0.5	2.3	4.7	2.7	4.5	4.8	6.5	3.6	4.9

Table 1

Supplementary Table 1: Detailed sleep-stage statistics of the individual subjects in the database. Inter-subject variability in the durations of sleep stages is typical, and does not affect the reported findings of distinct classes of brain wave interaction profiles, as shown by the consistency in individual subjects distributions in Supplementary Fig. 3 and supported by Wilcoxon Signed-rank tests (Materials and Methods, subsection Distribution profiles of cross-correlation values).
